# The Silk Route to Green Catalysis: Silk Fibroin as a Recyclable Ligand for Iron‐Catalyzed Olefin Epoxidation

**DOI:** 10.1002/cssc.202501841

**Published:** 2025-11-03

**Authors:** Carola Ricciardelli, Davide Blasi, Sabrina Bertini, Irene Tagliaro, Enrico Scelsi, Giuseppe V. Bianco, Elvira De Giglio, Pietro Cotugno, Gianluca M. Farinola

**Affiliations:** ^1^ Department of Chemistry University of Bari Aldo Moro Via E. Orabona 4 70125 Bari Italy; ^2^ Istituto di Ricerche Chimiche e Biochimiche G. Ronzoni 20133 Milan Italy; ^3^ Department of Materials Science University of Milano‐Bicocca 20125 Milan Italy; ^4^ Institute of Nanotechnology CNR‐NANOTEC Bari Division Via E. Orabona 4 70126 Bari Italy

**Keywords:** biopolymer, epoxidation reaction, iron catalysis, silk fibroin

## Abstract

A sustainable, efficient, and cost‐effective iron‐catalyzed olefin epoxidation is achieved by coordinating iron ions with silk fibroin (SF), a biocompatible protein derived from *Bombyx mori* cocoons. Unlike conventional systems based on complex ligands or synthetic supports, SF acts both as a support and as a recyclable ligand, efficiently coordinating iron through a simple aqueous process. The resulting SF–coordinated Iron system [Fe(SF)] promotes epoxidation of a broad range of olefins under mild conditions, with excellent yields and low metal loading. Notably, the system combines high activity with remarkable recyclability, and it can be easily regenerated. This work introduces a green, scalable strategy for iron catalysis, demonstrating the untapped potential of a natural polymer as a renewable ligand in heterogeneous catalysis.

## Graphical abstract

Iron coordinated on silk fibroin from *Bombyx mori* is easily prepared and catalyzes olefin epoxidation under mild, sustainable conditions with O_2_. With minimal iron loading, the catalyst is highly recyclable and fully regenerable, offering a green route to heterogeneous catalysis.

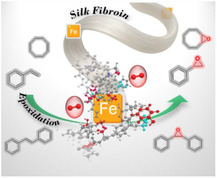

## 1 Introduction

Transition metal catalysis has revolutionized the chemical and pharmaceutical industrial production, thanks to the use of platinum group metals (PGMs) such as palladium, iridium, and platinum. These metals are essential for homogeneous catalysis and organic synthesis, but they also pose significant challenges due to their scarcity, high costs, and toxicity.^[^ ^1^, ^2^ ^]^ Actually, PGMs, along with other metals like nickel, copper, manganese, and titanium, are included in the European list of Critical Raw Materials, updated annually.^[^ ^3^ ^]^ Their long‐term sustainability is therefore under question, and research is increasingly focused on more environmentally friendly alternatives.^[^ ^4^, ^5^, ^6^ ^]^ Although catalyst recycling offers a potential solution, using more abundant and easily recyclable metals is often a convenient approach.^[^ ^7^ ^]^ Iron is abundant, inexpensive, and environmentally friendly, with redox and coordination properties that make it a promising substitute for precious metals in many processes.^[^ ^8^ ^]^ Natural systems, such as oxidative enzymes, demonstrate the catalytic potential of iron, inspiring the development of protein‐based iron catalysts, including both heme and nonheme complexes.^[^ ^9^ ^]^

Among all the possible fields of applications, olefin epoxidation is interesting from an industrial perspective, since epoxides are valuable intermediates in organic synthesis.^[^ ^10^, ^11^ ^]^

**Figure 1:**
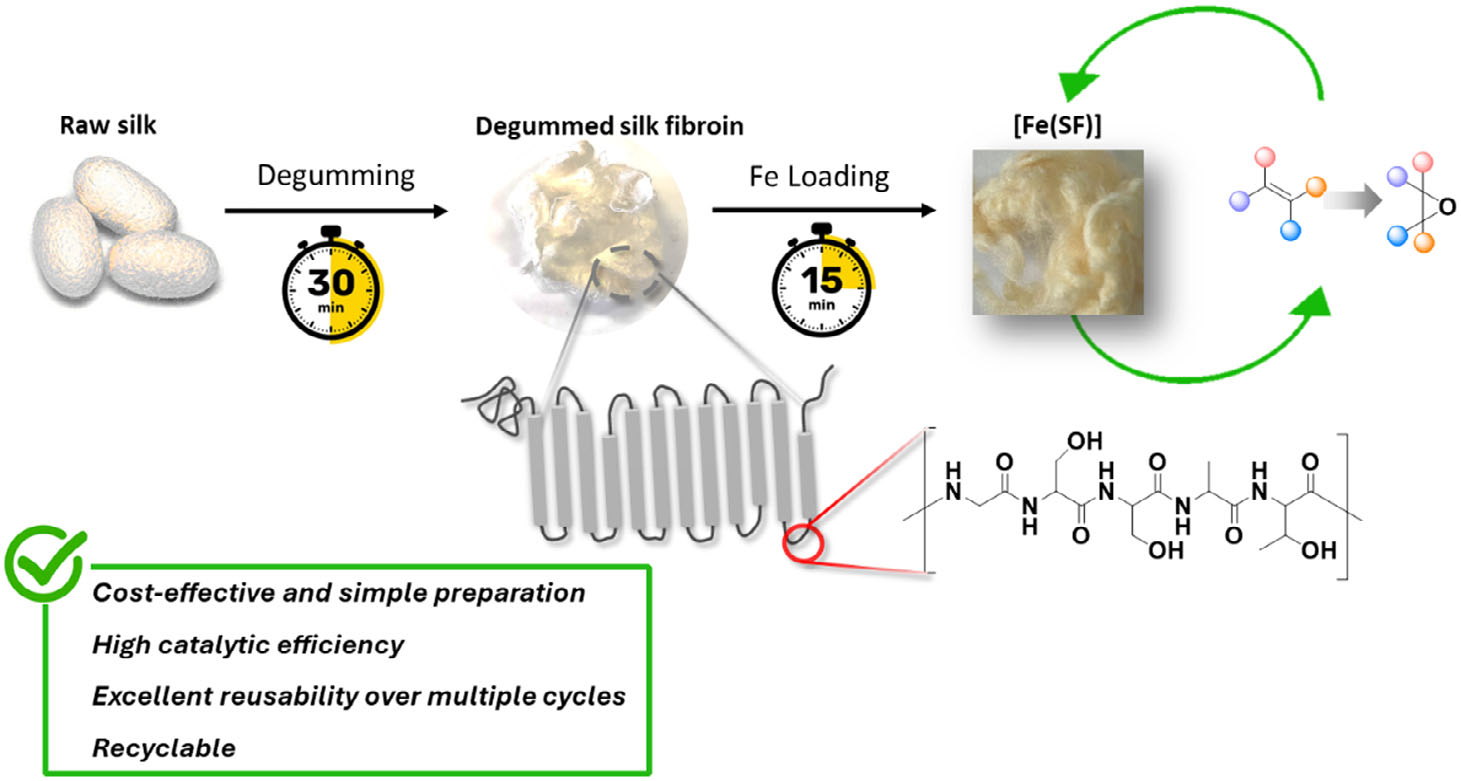
Preparation of the [Fe(SF)] catalyst, with a structural representation of the SF filament. The hydrophilic amorphous regions are highlighted in red.

Over the past decades, homogeneous iron catalysts have been extensively used for this reaction, thanks to their low cost and availability. Many Iron catalysts are based on small ligands, such as imidazole derivatives, pyridine‐2,6‐dicarboxylic acid (H_2_pydic), and benzylamines (**Figure** **1**). On the other hand, heterogeneous catalysts offer superior recyclability and efficiency and represent a valuable alternative for industrial applications.^[^ ^9^, ^12^, ^13^, ^14^, ^15^, ^16^ ^]^ Key advancements include the use of solid supports designed to form coordination bonds with metal species. Metal oxides, amorphous alumina‐supported FeOx, metal‐organic frameworks, covalent‐organic frameworks, and Fe‐incorporated carbon‐supported systems have been employed successfully for alkene epoxidation.^[^ ^17^, ^18^, ^19^, ^20^, ^21^, ^22^, ^23^, ^24^, ^25^ ^]^ However, supported catalysts preparation often involves multistep processes, making it crucial to explore more straightforward and efficient alternatives suitable for mass‐scale reactions.

In this context, an important issue concerns ligands: typically, ligands are not recoverable or recyclable, unlike the metals themselves. The complexity involved in synthesizing ligands and their limited recyclability represent significant drawbacks to large‐scale application.^[^ ^5^ ^]^

We report here an innovative approach that involves silk fibroin (SF) acting as a ligand for iron ions to catalyze epoxidation reactions. Silk, a natural biopolymer obtained from the cocoons of the silkworm *Bombyx mori*, is composed of hydrophobic crystalline domains and disordered hydrophilic regions.^[^ ^26^ ^]^ This unique structural organization endows SF with a combination of excellent mechanical properties, biocompatibility,^[^ ^27^, ^28^ ^]^ chemical stability, and versatility.^[^ ^29^, ^30^ ^]^ Notably, the presence of hydrophilic domains enables SF to coordinate metal ions, a feature that has already been exploited in the development of catalytic systems (Figure 1).^[^ ^31^ ^]^ In fact, SF has been successfully employed as environmentally friendly support for a variety of metal‐based catalysts, first for hydrogenation reactions^[^ ^32^, ^33^ ^]^ and more recently in cross‐coupling reactions.^[^ ^34^, ^35^, ^36^ ^]^ Moreover, the use of SF as support for an iron catalyst in phenol hydroxylation has been reported.^[^ ^37^ ^]^ We demonstrate here that SF can function not only as a passive support for a metal catalyst (Iron in this study) but also as an active ligand. This coordinating ability of SF opens up opportunities to enhance catalyst performance and assess new reactivity profiles in sustainable catalytic processes. A major advantage of this approach is that the [Fe(SF)] complex can be recovered and reused multiple times. Even after its catalytic activity declines, the SF ligand can be efficiently recycled and reused in the preparation of a fresh catalyst, making the process more sustainable and cost‐effective. This feature sets our protocol apart from other methods that rely on the complex synthesis of expensive ligands that are difficult to recover.

Building on these considerations, we have explored the interactions between iron ions and the biopolymer. The resulting catalyst, simply obtained by combining cost‐effective, commercially available, biocompatible sources, exhibits remarkable activity in epoxidation reactions, highlighting its promising potential in the field of sustainable chemistry.

## 2 Results and Discussion

### 2.1 Catalyst Preparation and Characterization

The catalyst [Fe(SF)] was obtained by soaking degummed SF fibers in a boiling aqueous solution of Fe(NO_3_)_3_·9H_2_O (6 × 10^−3^ M) for 15 min (vide infra). [Fe(SF)] fibers were then filtered and rinsed three times with distilled water, followed by a final wash with ethanol to remove any noncoordinated physiosorbed salt residue. The iron content was determined via inductively coupled plasma‐mass spectrometry (ICP‐MS) analysis, providing a value of 0.08 wt%, which represents a significantly lower loading compared to other supported iron catalysts reported in the literature,^[^ ^38^ ^]^ and provides the first indication that iron is not simply physiosorbed onto the biopolymer.

**Figure 2:**
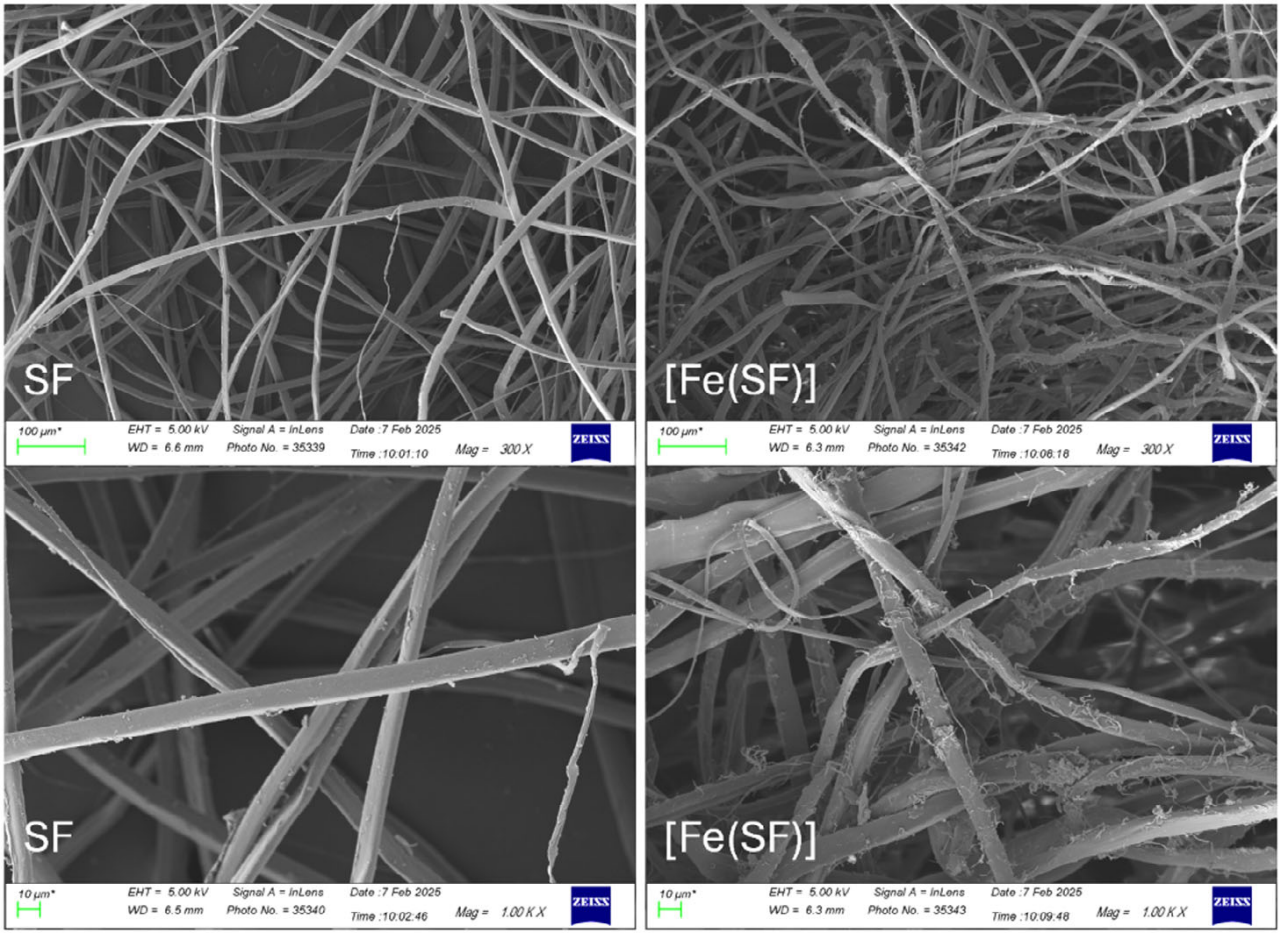
SEM images of degummed SF and [Fe(SF)] complex.

Scanning electron microscopy (SEM) images (**Figure** **2**) reveal that both the initial degummed SF and the [Fe(SF)] exhibit fibers with an average diameter of ≈10 μm, in line with our previous findings.^[^ ^34^, ^39^ ^]^ Notably, [Fe(SF)] fibers are entangled with thinner, shorter fibers (≈500 nm in diameter). Additionally, the surface of the thicker fibers in [Fe(SF)] appears rougher than in the pristine sample, with smaller fibers branching into shorter filaments.

Further characterization by attenuated total reflectance‐fourier transform infrared spectroscopy indicated that the secondary structure of the biopolymer remained unchanged after the treatment (Figure S1, Supporting Information). X‐ray diffraction analysis confirmed that the crystalline structure of the silk was not affected by the presence of Fe ions, suggesting their preferential localization in the amorphous regions and the absence of detectable Fe oxide phases (Figure S4, Supporting Information). A thermogravimetric analysis (TGA) was performed on both degummed SF and [Fe(SF)] to assess potential changes in terms of degradability after the treatment of the protein with the iron solution. The TGA profiles of both samples revealed no significant difference, with each displaying a single predominant degradation phase of the biopolymer at 326 °C. This suggests that the treatment with iron salt did not substantially alter the thermal stability or degradation behavior of the SF, indicating that its structural integrity remained largely unchanged after the treatment with the iron salt (Figure S6, Supporting Information).

X‐ray photoelectron spectroscopy (XPS) was used to investigate the potential binding sites between the protein and Fe atoms, as well as to determine the oxidation state of Fe. Surface atomic compositions of both SF and [Fe(SF)] samples, summarized in **Table** **1**, show the presence of Fe (2.4%) in the [Fe(SF)] sample, and confirm the metal incorporation.

**Table 1:** Surface atomic composition of SF and [Fe(SF)].

Samples	Surface atomic composition				
SF	C 69.1%	O 18.6%	N 10.0%	Si 1.7%	Ca 0.5%
[Fe(SF)]	C 60.1%	O 25.8%	N 10.3%	Fe 2.4%	Si 1.5%

**Figure 3:**
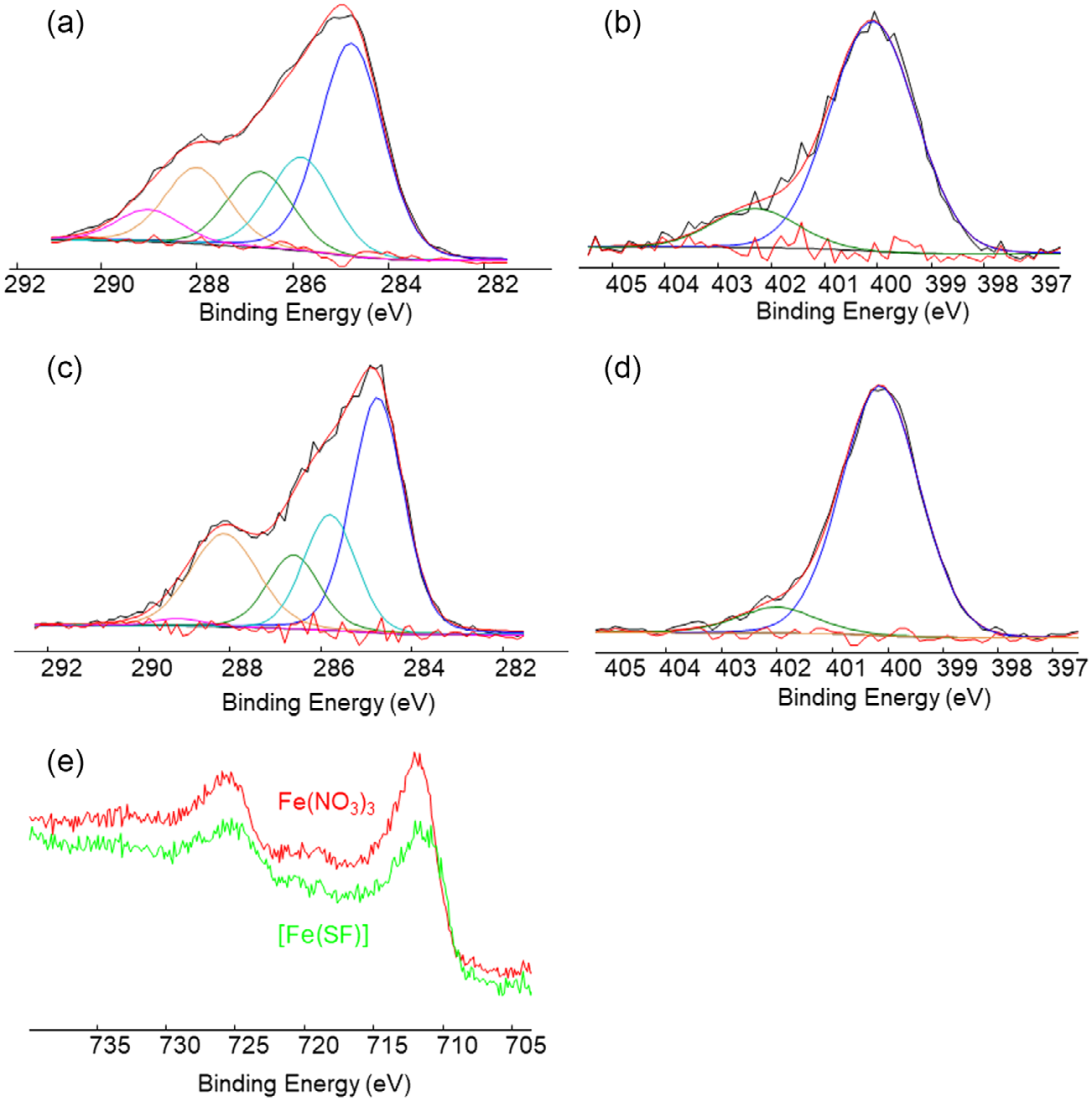
XPS high‐resolution spectra and relevant curve fitting of C1s a,c) and N1s b,d) regions recorded on SF (a,b) and [Fe(SF)] (c,d). In panel. In panel e) the comparison between Fe2p regions recorded on Fe(NO_3_)_3_ and [Fe(SF)] samples was shown.

In **Figure** **3**a–d, C1s (Figure 3a,c) and N1s (Figure 3b,d) high‐resolution spectra and relevant curve fittings, recorded on SF and [Fe(SF)] samples, were shown. Peak attributions, binding energies, and area percentages are reported in **Table** **2**. The deconvolution of the SF C1s spectrum (Figure 3a) was performed using five components relevant to the protein functional groups, but the component at 284.8 eV was ascribable to ubiquitous hydrocarbon contamination. In the C1s spectrum of [Fe(SF)] (Figure 3c), no additional contributions were detected and the only significant change resulted in a decrease in the carboxylic peak area and a proportional increase of the peak area at 288.1 eV (i.e., COOH/N–C=O peaks area ratios are equal to 0.42 and 0.06 in SF and [Fe(SF)], respectively). This finding could be explained by considering that iron ions likely interact with carboxylate‐containing amino acids located in the amorphous region of SF, shifting the contribution of these coordinated functionalities to lower binding energies similar to those observed for the amidic moieties. As far as the SF N1s signal is concerned (Figure 3b), the deconvolution was performed with two components, in perfect agreement with what was observed by Posada et al.^[^ ^40^ ^]^ The same components were also detected on the [Fe(SF)] catalyst with only a slight decrease in the –NH^+^ peak area percentage. Therefore, it can be hypothesized that the possible interaction between the iron ions and amide nitrogen does not lead to a significant change in the nitrogen binding energy, thus not causing a variation in the signal fitting.^[^ ^41^ ^]^ Furthermore, the comparison of Fe 2p spectra recorded for Fe(NO_3_)_3_ and [Fe(SF)] samples reveals nearly identical shapes, suggesting that the oxidation state of iron in the catalyst is predominantly Fe^3+^.

**Table 2:** C1s and N1s peaks attributions, binding energies (BE), and area percentages.

C1s Peaks’ Attributions	SF [BE/Area%]	[Fe(SF)] [BE/Area%]
CHx	284.8 eV (43.9%)	284.8 eV (42.7%)
C–NH	285.8 eV (19.4%)	285.8 eV (20.2%)
COH,C–NH^+^	286.7 eV (14.7%)	286.6 eV (13.9%)
N–C=O	288.0 eV (15.6%)	288.1 eV (21.9%)
O–C=O	289.0 eV (6.5%)	289.1 eV (1.4%)
N1s Peaks’ Attributions	SF (BE/Area %)	[Fe(SF)] (BE/Area%)
N–C=O	400.1 eV (85.4%)	400.1 eV (89.7%)
–NH^+^	402.2 eV (14.6%)	402.0 eV (10.3%)

**Figure 4:**
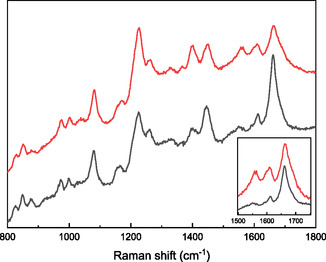
Raman spectra of SF (black) and [Fe(SF)] (red). Spectra in the inset are normalized to the maximum intensity of the 1660 cm^−1^ band.

Raman spectroscopy was used (**Figure** **4**) to support the XPS data suggesting interaction of Fe^3+^ ions with the carboxylate and possibly the amidic moieties in the amorphous regions of SF, and to explore the associated conformational changes upon metal coordination, Notably, the intrinsic fluorescence of SF caused by residual aromatic amino acids (mainly tyrosine) in SF remains unchanged after metal ion incorporation. Therefore, the spectra were not corrected by baseline subtraction, which could alter the relative intensities of Raman peaks with low signal. The spectra display the main Raman features of solid‐state SF, including bands associated with peptide linkages, i.e., the Amide bands: (i) the Amide I band at ≈1660 cm^−1^ (C=O stretching) (ii) the Amide II band at ≈1550 cm^−1^ (N–H bending), (iii) the complex Amide III region, with maxima around 1230 and 1270 cm^−1^ (mainly arising from C–N stretching).^[^ ^42^, ^43^ ^]^ After Fe^3+^ coordination, the Raman spectrum of [Fe(SF)] shows a reduced relative intensity of the Amide I band, along with a shift of its maximum toward higher wavenumbers. Moreover, the inset in Figure 4 (where the Amide I bands of SF and [Fe(SF)] are normalized to their maximum intensity) highlights a more pronounced shoulder at higher wavenumber in the [Fe(SF)] sample following metal ion coordination. These changes in the Amide I band are consistent with previous reports on metal ion incorporation into SF, attributed to coordination‐induced effects on protein folding.^[^ ^44^ ^]^ The correlation between Amide band features (frequency, relative intensity, broadening, etc.) and the secondary structure of SF arises from distinct hydrogen‐bonding patterns in α‐helix, β‐sheet, or disordered structures. Specifically, Fe^3+^ coordination has been reported to induce a conformational transition from α‐helix (characteristic Amide I band at ≈1650 cm^−1^) to β‐sheet (≈1670 cm^−1^),^[^ ^45^ ^]^ which results in the observed decrease in intensity and broadening toward higher wavenumbers in the [Fe(SF)] Amide I band. Similarly, the intensity ratio between the Amide III peaks at 1230 and 1270 cm^−1^ increases, indicating a higher contribution of the characteristic β‐sheet mode (1229 cm^−1^).^[^ ^46^ ^]^ Interestingly, significant changes are also observed in the Amide II band (1550 cm^−1^), which mainly derives from N–H bending and is typically less sensitive to secondary structure than the Amide I band.^[^ ^46^, ^47^ ^]^ This typically weak Raman mode appears well‐defined in the [Fe(SF)] spectrum. Such enhancement can be related to a strong involvement of NH groups in Fe^3+^ coordination. Finally, an increase in protein structural order is supported by the rise in the intensity ratio between the δCαH_2_ and δCH_2_ Raman modes (located at 1400 and 1450 cm^−1^, respectively).^[^ ^46^ ^]^ The bands at 1081 and 870 cm^−1^ (related to νC–C skeletal and νC–N vibrations), as well as the Raman peaks associated with tyrosine residues (1512, 998, 850, and 820 cm^−1^), do not show noticeable changes upon metal ion coordination.^[^ ^46^ ^]^

In order to further investigate whether Fe^3+^ coordination affects the macromolecular properties of the protein matrix, high‐performance size exclusion chromatography with triple detection array (HP‐SEC‐TDA) was performed. Therefore, HP‐SEC‐TDA with multidetector systems (Right Angle and Low Angle Light Scattering, Refractive Index and Viscosimeter), was used to determine the molecular weight distribution, intrinsic viscosity, hydrodynamic radius and Mark–Houwink parameters of the [Fe(SF)] complex. The samples had an elution volume between 10 and 16 mL with a broad bell‐shaped chromatographic peak, caused by a high polydispersity index (Figure S5, Supporting Information). Mw (weight average molecular weight), Mn (number average molecular weight), polydispersity (expressed as Mw/Mn ratio), *μ* (intrinsic viscosity), Rh (hydrodynamic radius) and *a*, corresponding to the slope of the Mark–Houwink curve, are reported in **Table** **3**. All the results refer to the mean values of duplicate injections.

**Table 3:** HP‐SEC‐TDA results of SF and [Fe(SF)].

Sample	Mw [kDa]a)	Mn [kDa]b)	Pd [Mw/Mn]c)	[*h*] [dl g^−1^]d)	Rh [nm]e)	*a* f)
SF	173	133	1.3	0.4	9.8	0.49
[Fe(SF)]	282	233	1.2	0.2	9.0	0.65

a) Weight average molecular weight;

b) Number average molecular weight;

c) Polydispersity;

d) Intrinsic viscosity;

e) Hydrodynamic radius;

f) Mark–Houwink parameter.

SF Mw = 173 kDa and Mn = 133 kDa were measured, respectively; [Fe(SF)] complex showed higher molecular weight values with respect to the pristine SF, which is compatible with the incorporation of iron atoms into the SF chain without determining any depolymerization of the biopolymer. The Mw/Mn increases compared to the pristine SF, meaning an increase in polydispersity after the complexation. The hydrodynamic radius (Rh) for SF and [Fe(SF)] remains in the same range, indicating a stable configuration of protein. The intrinsic viscosity, [*η*], of [Fe(SF)] is lower compared to SF and the [*η*] decreases with the Fe concentration. This is likely due to an increase of structural density caused by the introduction of iron ions, which causes a decrease in viscosity. The Mark–Houwink parameter, *a*, is similar for all the samples and comparable to data reported in the literature.^[^ ^48^ ^]^ The *a* values are in the range of 0.5–0.6 thus indicating that, regardless of Fe, the protein remains in a coiled structure.

### 2.2 Epoxidation of Styrene

The epoxidation of styrene was chosen as a model reaction to investigate the catalytic activity of the Fe‐coordinated by SF. The reaction carried out in acetonitrile (ACN), in the presence of [Fe(SF)] as the catalyst and cyclohexanecarbaldehyde (cyCHO) as co‐reductant with oxygen as the oxidant, afforded the epoxide. The reaction was regularly monitored by gas chromatography‐mass spectrometry (GC‐MS), and the results are summarized in **Table** **4**. Four sets of reference experiments were performed: (i) with SF in the absence of iron catalyst, (ii) with iron nitrate only (the salt used for the preparation of the catalyst) without SF, (iii) with SF without iron, and (iv) introducing both the SF and iron nitrate directly in the reaction mixture. In all these experiments, the epoxide was not detected in a significant amount (Table 4, entries 1–4). The choice of the co‐reductants largely affects the reaction outcome, and the reaction does not proceed in the absence of any aldehyde (Table 4, entry 6). In the oxygen environment, the aldehyde is oxidized in situ to form the corresponding peracid, which acts as the active oxidizing agent. This peracid promotes the conversion of styrene to styrene oxide, with the simultaneous formation of carboxylic acid as a byproduct.^[^ ^49^ ^]^ The bold entry in Table 4 indicates the best operating conditions.

**Table 4:** Optimization of epoxidation reaction.

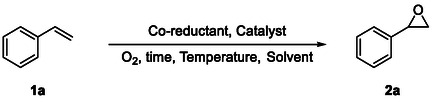					
Entry	Co‐reductant	Catalyst	*T* [°C]	*t* [h]	Yield of 2a [%]
1	CyCHO	–	80	6	Traces
2	CyCHO	Fe(NO_3_)_3_	80	6	Traces
3	CyCHO	SF	80	6	Traces
4	CyCHO	Fe(NO_3_)_3_ + SF	80	6	5
5	CyCHO	[Fe(SF)]	80	6	90
6	–	[Fe(SF)]	80	6	3
7	PivCHO	[Fe(SF)]	80	6	64
8a)	H_2_O_2_/CyCHO	[Fe(SF)]	80	6	6
9b)	CyCHO	[Fe(SF)]	80	6	60
10c)	CyCHO	[Fe(SF)]	80	6	80
11	CyCHO	[Fe(SF)]	60	6	Traces
12	CyCHO	[Fe(SF)]	70	6	63
**13**	**CyCHO**	**[Fe(SF)]**	**80**	**3**	**88**
14	CyCHO	[Fe(SF)]	80	2	60
15d)	CyCHO	[Fe(SF)]	80	3	84
16e)	CyCHO	[Fe(SF)]	80	3	56
17	CyCHO/BHT	[Fe(SF)]	80	6	n.d.

Reactions were performed under the following conditions: styrene (1 mmol), co‐reductant (1.5 mmol), 2 mL of ACN as solvent, 25 mg of catalyst, under an oxygen atmosphere. Yields determined by gas chromatography with flame ionization detection (GC‐FID);

a) Reactions performed under air;

b) Reaction conducted with 1.1 mmol of cyCHO;

c) Reaction conducted with 2.0 mmol of cyCHO;

d) Reaction conducted with 50 mg of [Fe(SF)];

e) Reaction conducted with 12 mg of [Fe(SF)].

Tests carried out with other possible co‐reductants (cyclohexanecarboxilic acid, *tert*‐butyl hydroperoxide) yielded no product formation (Table S1, Supporting Information). Among the aliphatic aldehydes tested, pivalaldehyde afforded the epoxide, but in relatively low yield (Table 4, entry 7). In contrast, cyCHO was found to be the most effective co‐reductant. Hydrogen peroxide was also tested as an oxidant in the presence of the co‐reductant aldehyde, resulting in a very low yield (Table 4, entries 8). The effect of the cyCHO/styrene molar ratio on epoxidation was then examined. As shown in Table 4, styrene conversion increased from 60% to 88% when the ratio rose from 1.1:1.0 to 1.5:1.0 (entries 5, 9). However, a further increment of the aldehyde ratio (entry 10) did not significantly enhance the epoxidation reaction and made purification more difficult due to the higher concentration of co‐reductant. Based on these findings, a molar ratio of 1.5:1.0 was identified to be the optimal condition for this study.

Lower temperatures were tested, but lower yields were found (Table 4, entries 11–12). The influence of different solvents on the reaction outcome was systematically evaluated (Table S1, Supporting Information). No formation of the desired product was observed in any of the tested solvents, except acetonitrile, which yielded good results in the same conditions. The role of acetonitrile as a co‐reactant in the oxidation reaction through the formation of peroxyimidic acid was ruled out by NMR spectroscopy, which showed the absence of any MeCONH_2_ detected as a byproduct in the catalytic oxidation.^[^ ^50^ ^]^

A 3 h reaction time was set to investigate the best catalyst loading (Table 4, entries 13,14). Notably, increasing the amount of [Fe(SF)] from 25 mg to 50 mg did not result in any enhancement of the catalytic activity. Conversely, reducing the amount to 12 mg led to a decrease in performance (Table 4, entries 15, 16). In fact, the minimal amount of [Fe(SF)] needed to obtain optimal results was found to be 25 mg, which corresponds to an iron loading of 0.08 wt% with respect to the starting styrene. To provide a broader context, Table S2, Supporting Information, lists selected examples of metal‐based catalysts, including commercially available ones, reported for alkene epoxidation, which typically require higher metal loadings than the present system.^[^ ^51^, ^52^, ^53^, ^54^, ^55^, ^56^, ^57^, ^58^, ^59^, ^60^ ^]^

To explore the mechanism of epoxidation, butylated hydroxytoluene (BHT), known for its role as a quencher of reactive oxygen species, was introduced into the reaction system. The absence of the epoxidation product in the presence of BHT strongly suggests that the reaction proceeds via a radical mechanism (Table 4, entry 17).^[^ ^61^ ^]^ The optimal reaction conditions are represented by entry 13.

To elucidate the oxygen source for the epoxidation, an ^18^O‐labeling experiment was performed using an ^18^O atmosphere. GC‐MS analysis of the resulting styrene oxide revealed the presence of ^18^O, indicating that the oxygen atom in the epoxide originates from molecular oxygen.

### 2.3 Substrate Scope

**Scheme 1:**
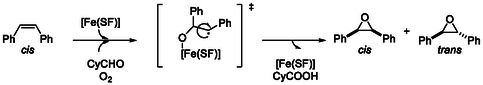
Plausible reaction scheme for the oxidation of *cis*‐stilbene.

Various olefins, including terminal, cyclic, and aromatic olefins, were oxidized in the optimized conditions, with molecular oxygen in the presence of [Fe(SF)], affording the corresponding epoxides with high yields (**Table** **5**), and fully characterized by NMR spectroscopy (details in SI). The epoxide is the main product in the oxidation of styrene and its derivatives; however, a small amount of the corresponding benzaldehydes is also formed (2a–e). Benzaldehydes are likely generated by bond breaking of the plausible intermediate benzyl radical. Cyclooctene was converted to its epoxide by 97% after 2 h (2f). *cis*‐Stilbene is a frequently used substrate for the study of olefin epoxidation mechanism, as the ratio of *cis*‐ and *trans*‐isomers in the resulting stilbene oxides provides mechanistic insights.^[^ ^62^ ^]^ In our study, the oxidation of *cis*‐stilbene produced both the *cis*‐ and *trans*‐stilbene oxide with a molar ratio of 25:75 (2 g). This result supports the hypothesis that the oxidation reaction proceeds through a benzyl radical intermediate, which is sufficiently stable to allow free rotation about the C—C bond axis. The formation of *trans*‐stilbene oxide as the main product from both *cis*‐ and *trans*‐stilbenes via a radical intermediate is expected, due to the higher thermodynamic stability of the trans in comparison to the cis stereoisomer, in which the phenyl groups are located in antiposition with respect to each other (**Scheme** **1**).^[^ ^63^, ^64^ ^]^

**Table 5:** Substrate scope of the [Fe(SF)] catalyzed epoxidation reactiona).

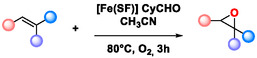
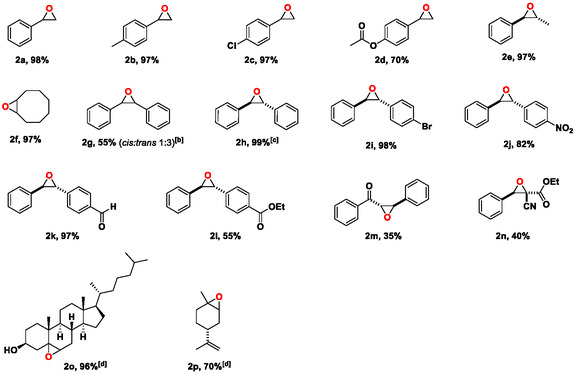

a) Reactions were performed using 1 mmol of olefin 1, 1.5 mmol of cyclohexanecarbaldehyde 2, 0.08 % of [Fe(SF)], 2 mL of ACN, for 3 h under an oxygen atmosphere. Yields refer to isolated products characterized by NMR spectroscopy.

b) *Cis*‐stilbene used as substrate;

c) *Trans*‐stilbene used as substrate;

d) Yield after 24 h.

*trans*‐Stilbene was oxidized quantitatively by [Fe(SF)] to the corresponding epoxide with 100% stereoselectivity (2h). The same selectivity is also achieved by substituted stilbenes with a yield up to 55% (2i–l). α,β‐Unsaturated systems, such as chalcones and esters, were also tested. However, in these cases, the corresponding epoxides were obtained with lower yields, probably due to the steric hindrance associated with the double bond (2m‐n). The protocol was further extended to cholesterol, as an example of a more complex substrate, achieving complete conversion to the corresponding epoxide after extending the reaction time to 24 h. Both isomers, cholesterol‐5α,6α‐epoxide and cholesterol‐5β,6β‐epoxide, were obtained with a ratio of 1.0:1.9 (2o). Limonene was also successfully converted into its corresponding epoxide (2p), a product that has been considered a promising building block in the production of biopolycarbonates and nonisocyanate polyurethanes, due to its attractive thermal and optical properties.^[^ ^65^, ^66^ ^]^

### 2.4 [Fe(SF)] Catalyst Recycling

**Figure 5:**
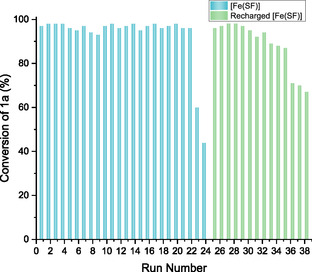
Recycling tests of [Fe(SF)] promoted epoxidation, performed under the optimized conditions. Conversion of 1a (styrene) was estimated by GC‐FID analysis of the crude product.

The stability and reusability of the [Fe(SF)] catalyst were evaluated in the epoxidation of styrene through recycling experiments. After each cycle, the catalyst was readily recovered by simple filtration and washed with acetonitrile and ethanol, and then reused for a further epoxidation reaction under the optimized conditions. The catalyst maintained its activity over 20 consecutive cycles without any significant loss in performance. A noticeable drop in conversion to 40% was observed only after the 23rd cycle, indicating a gradual decrease in catalytic activity (**Figure** **5**). A distinctive advantage of this catalytic system is its easy and effective recovery. Actually, the catalyst resulting from the 25th run we simply recharged by immersion in a boiling aqueous solution of iron nitrate for 15 min, using the same protocol as the initial preparation. This easy recharging protocol fully restores the catalytic performance, affording complete conversion in the subsequent reaction cycles, up to (at least) eight additional recycling runs without any detectable decline in activity.

These results show not only the remarkable stability of the [Fe(SF)] catalyst but also highlight the possibility to readily restore its catalytic activity, which is an additional advantage in catalyst sustainability. Unlike many systems where catalyst recycling often refers solely to the metal component, our approach demonstrates the effective recycling of both the metal complex [Fe(SF)] and, crucially, the ligand scaffold (SF), thereby minimizing waste and maximizing resource efficiency.

To quantify the iron leached into solution, samples were collected after the 1st and 24th reaction cycles. The reaction mixtures were filtered using a 0.2‐micron Teflon filter, and the resulting clear solutions were mineralized with aqua regia. ICP analysis was performed to measure iron content, revealing levels of 22.5 ppb (0.004 wt%) after the 1st cycle and 47.0 ppb (0.008 wt%) after the 24th. It is important to note that iron is a ubiquitous element, and these low values are not directly attributable to catalyst leaching. In fact, ICP mass analysis of pure SF revealed an iron content of 19 ppm, further suggesting that the detected iron levels may not be solely a result of leaching from the catalyst. Nevertheless, the slight increase in iron content observed after the 24th cycle may indicate a minor metal loss during the recycling process, which could contribute to the drop in catalytic activity observed after the 22nd reuse. Hence, to assess the heterogeneity of the catalysis, Mailists' hot filtration test was performed. Two epoxidation reactions on styrene as substrate were carried out under the optimized conditions (Table 4, entry 13) and stopped after 2 h. For one of them, a 49% yield was found. For the other, the hot mixture was filtered to remove the catalyst, and the resulting solution was stirred again at 80 °C for 1 h, and the yield was found again at 49%. These results, along with the nonsignificant catalyst leaching observed, seem to confirm the heterogeneous nature of the catalytic system.

## 3 Conclusion

The remarkable chemical, thermal, and mechanical stability of SF fibers has been exploited to develop a new robust and versatile platform for heterogeneous catalysis. Iron was efficiently coordinated to the SF fibers through a simple, one‐step process in water, yielding a highly active catalyst for olefin epoxidation. A wide range of terminal, cyclic, and aromatic olefins was converted to the corresponding epoxides in good to excellent yields under mild conditions, using O_2_ as the oxidant and a minimal iron loading. In addition to its high catalytic activity, the catalyst demonstrated excellent stability, maintaining its performance over twenty recycling cycles, and decreasing to 40% only after the 24th cycle. Notably, catalytic activity was fully restored from the 25th cycle by easily soaking the catalyst in aqueous iron nitrate solution. This straightforward recharging strategy fully restored catalytic performance, providing complete conversion in subsequent reaction cycles. This is a significant result considering the limited recyclability typically associated with biopolymers and many synthetic supports used in epoxidation reactions. This study demonstrates that operatively simple epoxidation reactions can be performed without the need for complex and costly ligands for metal catalysts, which are often challenging to prepare. This work expands the application potential of the [Fe(SF)] system within the field of catalysis, and we aim to further explore its use in other catalytic reactions.

## Conflict of Interest

The authors declare no conflict of interest.

## Supporting information

Supplementary Material

## Data Availability

The data that support the findings of this study are available in the supplementary material of this article.
