# Supplementary material for: The Silk Route to Green Catalysis: Silk Fibroin as a Recyclable Ligand for Iron‐Catalyzed Olefin Epoxidation
Source: ChemSusChem. 2025 Nov 3;19(1):e202501841. doi: 10.1002/cssc.202501841 (PMC12767277; doi:10.1002/cssc.202501841)
Supplement: Supplementary file 1 — Supplementary Material [file CSSC-19-e202501841-s001.pdf]

Supporting Information

The Silk Route to Green Catalysis: using Silk Fibroin as a Recycle Ligand for Iron Epoxidation

Carola Ricciardelli,[a] Davide Blasi,[a] Sabrina Bertini,[b] Irene Tagliaro,[c] Enrico Scelsi,[a] Giuseppe V. Bianco,[d] Elvira De Giglio,[a] Pietro Cotugno*[a] Gianluca M. Farinola[a]

[a] Dr. C. Ricciardelli, Dr. D. Blasi, E. Scelsi, Prof. E. De Giglio, Prof. P. Cotugno, Prof. G. M. Farinola
Department of Chemistry
University of Bari Aldo Moro
Via E. Orabona 4, 70125 Bari, Italy
E-mail: pietro.cotugo@uniba.it

[b] Dr. S. Bertini Istituto di Ricerche Chimiche e Biochimiche G. Ronzoni
Milan 20133, Italy.

[c] Dr. I. Tagliaro
Department of Materials Science
University of Milano-Bicocca
Milan 20125, Italy

[d] Dr. G. V. Bianco
Institute of Nanotechnology
CNR-NANOTEC, Bari Division
Via E. Orabona 4, 70126 Bari, Italy

Experimental Section

General Information

Silk fibroin (SF) was obtained from *Bombyx mori* cocoons from Tajima Shoji (Japan). All the chemicals were purchased from commercial sources and used as received without purification. Column chromatographies were performed with Fluka silica gel, pore size 60 Å, 70-230 mesh, 63-200 µm. ^{1}H-NMR and ^{13}C-NMR spectra were recorded at room temperature in deuterated solvents solution with an Agilent 500 spectrometer, operating at a frequency of 500 MHz for ^{1}H, 125 MHz for ^{13}C; chemical shifts (δ) values are given in parts per million (ppm) and coupling constants (J) in Hertz. GC analyses were performed on an Agilent 7890B equipped with a Agilent J&W 5MS capillary column (30 m×0.25 mm id). GC-MS analyses were performed on a Thermo Polaris Q spectrometer equipped with a Macherey-Nagel Optima-1 capillary column (30 m×0.25 mm id), ionization mode EI (70 eV).

Preparation and Characterization of [Fe(SF)] catalyst

Preparation

Raw silk cocoons (5.00 g) were shredded in 1 cm pieces and boiled in a 0.02 M Na_2CO_3 aqueous solution (2L) at 100°C for 30 minutes under constant stirring to remove the external sericine coating. Degummed silk, called silk fibroin (SF) was washed in bidistilled water to remove the salts and the sericines extracted. The final SF was air-dried for 24 hours, obtaining 3.75 g of pure SF.[1] After the degumming process, the SF fibers were soaked in a boiling solution of $(FeNO_3)_3$ $9H_2O$ ($6{\cdot}10^{-3}$ M) for 15 minutes to give [Fe(SF)] fibers. The fibers were then filtered and rinsed three times with distilled water, followed by a final wash with ethanol to remove any salt residues. The pH of the $Fe(NO_3)_3 9H_2O$ solution before the addition of SF is 2.4 ± 0.2, while after removing the SF it decreases to 1.8 ± 0.2.

Characterization

ATR-FTIR analyses

ATR-FTIR spectra were acquired with a Perkin Elmer Spectrum Two Spectrophotometer equipped with a 2x2 mm Diamond crystal. Spectra were recorded in the range 4000 - 400 cm^{-1} with a 2 cm^{-1} resolution, using 0.25 cm^{-1} acquisition interval and acquiring 32 scans for each sample.

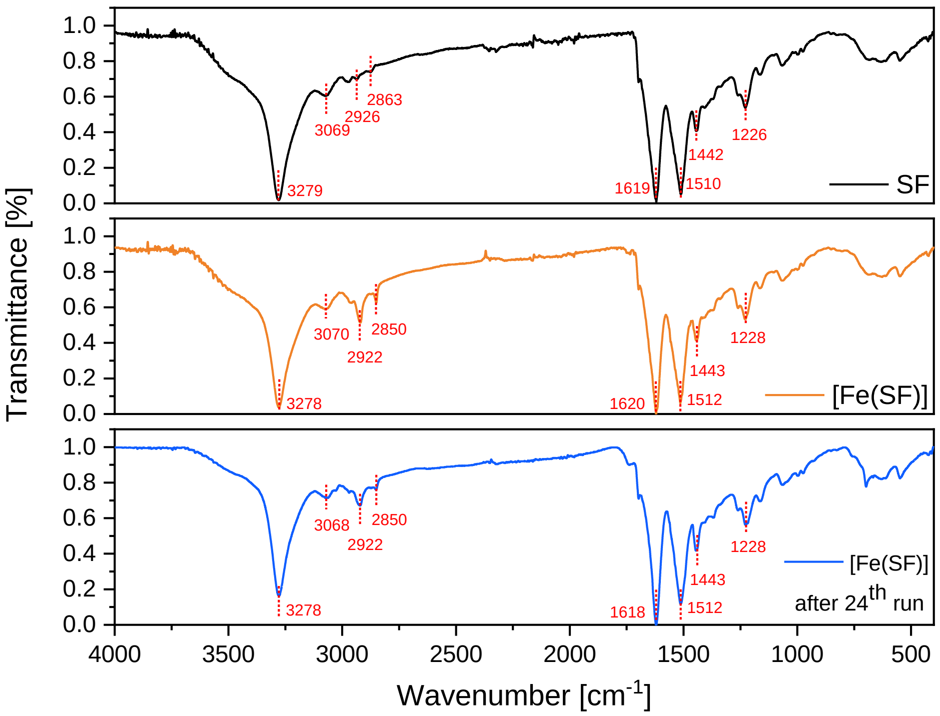

Figure S 1. ATR-FTIR Spectra of degummed SF fibers, [Fe(SF)] and [Fe(SF)] catalyst after 24th recycling runs. Red dash lines highlight the presence of typical SF bands and relative wavenumbers.

SEM images

The samples are analyzed by means of Scanning Electron Microscopy (SEM) with a Zeiss Gemini 500 Field-Emission SEM (Carl Zeiss Microscopy, Oberkochen, Germany) at 5 kV. The surfaces have been sputtered with 10 nm gold particles to enhance conductivity.

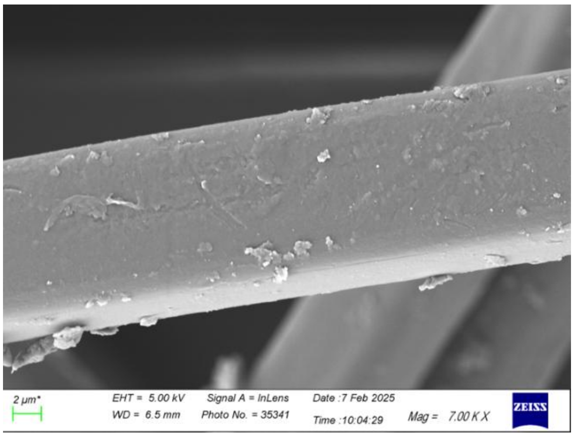

Figure S 2. SEM of Silk Fibroin at 7000x magnifications. Single fibers appear with a smooth surface and a squared section, with an average diameter of around 10 µm.

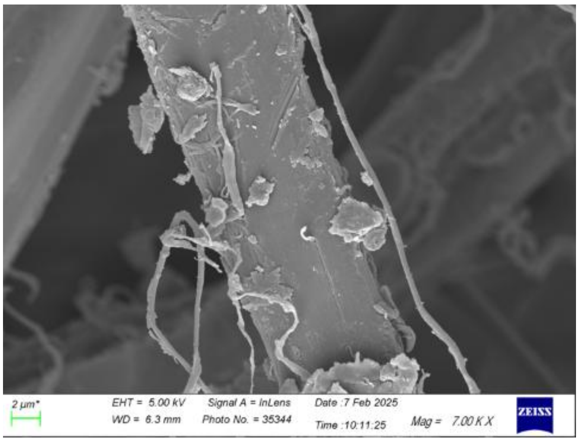

Figure S 3. SEM of [Fe(SF)] at 7000x magnifications. The surface of thicker fibers looks less smooth compared to the pristine sample, comprising smaller fibers branching out as shorter filaments.

XRD analyses

X-Ray diffraction analysis (XRD) was performed with a Rigaku Miniflex 600, X-ray diffractometer with Bragg-Brentano (θ - 2θ) parafocusing geometry. The instrument is operated at the maximum power 600 W (40 kV – 15 mA). The samples were compacted on the sample holder by placing a weight on top for 30 minutes. The acquisition was performed from 5◦ to 60◦ at a speed of 2◦/min with steps of 0.02◦.

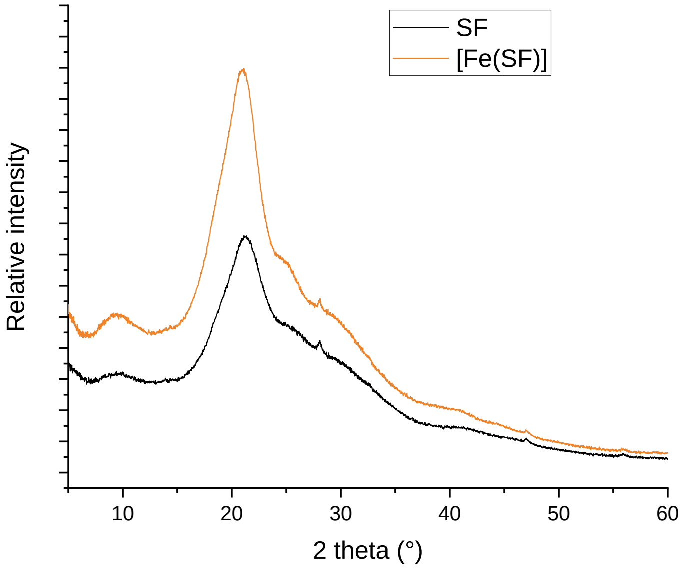

Figure S 4: 3 XRD of degummed Silk Fibroin (black), [Fe(SF)] (orange).

All samples show a partially amorphous structure together with crystalline typical patterns of silk II crystal with broad peaks at 9-10° and 20-21° 2θ, and to silk I with the shoulder peak at 25° 2θ.[2-3] From the comparison of the three diffractograms, it is clear that the crystalline structure is not affected by the presence of Fe ions. It is, therefore, possible to speculate that the presence of Fe ions may be preferentially located in the amorphous structure. Moreover, no signs of Fe oxides are detectable in the three samples.

Sample preparation for HP-SEC-TDA

The samples were dissolved in 9.3 M LiBr water solution (25%, w/v) at 60◦C for 4 h and filtered with 0.22 µm filter to eliminate impurities.

Molecular weight distribution by size exclusion chromatography with triple detector array (HP-SEC-TDA)

Chromatographic acquisitions were performed on Viscotek system model TDA302 (Malvern Panalytical, UK) equipped with a triple detector array exploiting simultaneous action of refraction index detector (RI), Right and Low Angle Light Scattering (RALS and LALS) and Viscometer (DP).

Measurements were performed at 40°C using 2 x TSKGMPWXL colums,13 µm, 7 mm ID x 30 cm L, in series (Tosoh Bioscience, Tokio, Japan). Urea 4M+0.05% NaN_3, prefiltered (0.22 µm Mixed cellulose Ester filter), was used as mobile phase at a flow rate of 0.6 mL/min. Chromatographic profiles were elaborated using the OmniSEC software version 4.6.2. The dn/dc value equal to 0.185, typical for proteins, was used.

The detectors were calibrated with Pullulan standard, with certified molecular weight, polydispersion index and intrinsic viscosity (PolyCAL-PullulanStd-102K Malvern Panalytical, UK). Samples were analysed at about 3-4 mg/mL solubilized as reported in Sample preparation

HP-SEC-TDA with multi-detector systems (Right Angle and Low Angle Light Scattering, Refractive Index and Viscosimeter), was used to determine the molecular weight distribution, intrinsic viscosity, hydrodynamic radius and Mark-Houwink parameters of the samples. After various preliminary tests, 4M Urea with 0.05% NaN_3 was chosen as mobile phase. The samples had an elution volume between 10 and 16 mL with a broad bell-shape chromatographic peak, caused by a high polydispersity index (Figure S 5). Mw (weight average molecular weight), Mn (number average molecular weight), polydispersity (expressed as Mw/Mn ratio), µ (intrinsic viscosity), Rh (hydrodynamic radius) and a, corresponding to the slope of the Mark-Houwink curve, are reported in Table 3. All the results refer to the mean values of duplicate injections.

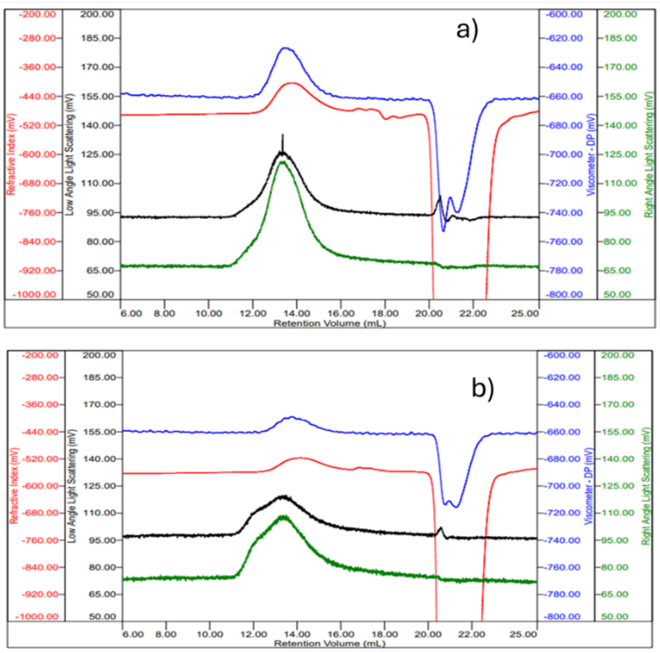

Figure S 5: Chromatographic profile (red—refractive index; black—low laser light scattering; green—right angle light scattering; blue—viscometer) of SF (a) [Fe(SF)](b).

Photon Correlation Spectroscopy and Zp

The PCS and Zp of Silk-fibroin compounds were measured using the Zetasizer Nano ZS (Malvern Panalytical, UK) with scattering angle of 173° and a 633-nm helium–neon laser. Data were analyzed using Zetasizer software version 7.11 (Malvern Panalytical, UK). For Size and Zp measurements, the sample solutions, prepared as reported in Sample preparation for HP-SEC-TDA, were diluted in deionized water to the desired concentration (about 2 mg/L). The Size measurement was performed at 20°C, 10 measurements, 10 run, 5 s time delay and general-purpose method for the acquisition using disposable folded capillary cells (ZEN0040, Malvern Panalytical, UK). The Zp measurement was performed at 20°C, 5 measurements, 10 run, 60 s time delay and monomodal purpose method for the acquisition using disposable folded capillary cells (DTS1070, Malvern Panalytical, UK).

TGA

Thermal gravimetric analysis (TGA) was carried out on a Pyris 1-Perkin Elmer thermogravimetric analysis system, applying a nitrogen flow of 40 mL/min. SF and [Fe(SF)] were maintained at 100°C for 3 min and then they were heated at a rate of 5°C min^{-1} in the range of temperature 100-400°C.

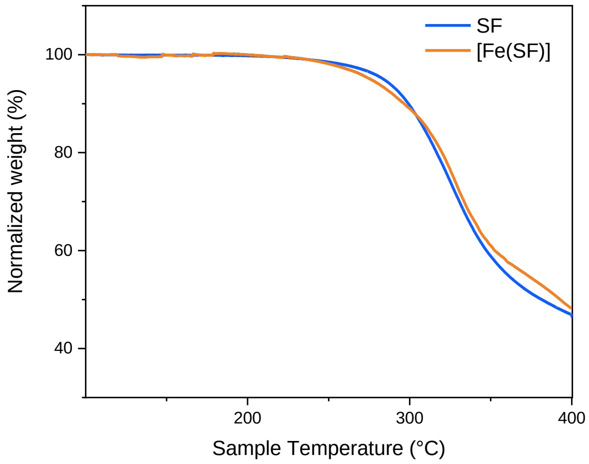

Figure S 6: TGA profiles of SF and [Fe(SF)]

ICP-MS

To determine the wt % of iron of the catalyst [Fe(SF)], an ICP-MS analysis was conducted using a Thermo Scientific iCAP Q spectrometer. This analysis followed a microwave-assisted mineralization of the sample, carried out in a Microsynth Milestone FKV Advanced Microwave Synthesis Labstation. Specifically, [Fe(SF)] (40 mg) was digested using 9 mL of HCl and 3 mL HNO_3. The temperature program applied consisted of two cycles, each lasting 15 minutes. In the first cycle, the vessel temperature was ramped up to a maximum of 200°C over 15 minutes. In the second cycle, the temperature was maintained at 200°C for an additional 15 minutes. After digestion, the resulting solution was then diluted with deionized water (13 mL) and used for ICP-MS analysis to determine the iron content. Limits of quantification were in the range of 0.2–6.7 ng/mL and the extraction recoveries varied in the range from 88 % to 110 %.

[Fe(SF)] catalyzed epoxidation reaction

General procedure

The epoxidation of olefines with O_2 was conducted in a 25-mL round-bottom flask. In a typical experiment, the flask was charged with substate (1 mmol), 2 mL of acetonitrile, 1.5 mmol of cy-CHO, and 25 mg of catalyst [Fe(SF)]. The flask was conditioned by purging with oxygen for 3 minutes and subsequently re-filled with oxygen. The reaction mixture was stirred at 80 °C for 3 h. At appropriate intervals, aliquots were removed and analyzed by GC-MS and GC-FID. After cooling to room temperature, the reaction mixture was diluted with H_2O and EtOAc and filtered. Filtrate was then extracted with EtOAc, washed with $NaHCO_3$, dried over anhydrous Na_2SO_4 and the solvent was removed under vacuum. The crude products, if needed, were purified though chromatographic columns, using different mixtures of organic solvents depending on the polarity of each compound. The acquired ^{1}H and ^{13}C spectra of all the synthesized compounds were compared with literature data and found to be in agreement. All experiments were carried out in triplicate. The reported conversions and yields represent the average values, with a standard deviation below 4 %.

The ^{18}O-labeling experiment was conducted following the procedure described above, except that the reaction flask was conditioned by purging with $^{18}O_2$. The reaction was monitored at different time intervals by GC-MS, and analysis of the resulting styrene oxide confirmed the incorporation of ^{18}O.

Table S1: Optimization of epoxidation reaction

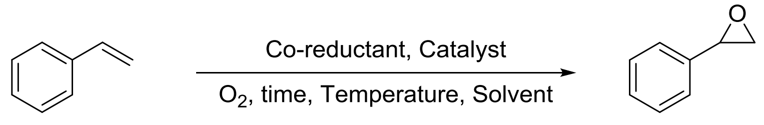

1a 2a

Entry	Co-reductant	Catalyst	Solvent	T (°C)	t (h)	Yield of 2a(%)
1	CyCOOH	[Fe(SF)]	ACN	80	6	n.d.
2	PivOOH	[Fe(SF)]	ACN	80	6	n.d.
3	BenzCHO	[Fe(SF)]	ACN	80	6	n.d.
4	Vanilline	[Fe(SF)]	ACN	80	6	n.d.
5[a]	H_2O_2	[Fe(SF)]	ACN	80	6	8
6	CyCHO	[Fe(SF)]	DMSO	80	6	n.d.
7	CyCHO	[Fe(SF)]	EtOH	80	6	n.d.

8	CyCHO	[Fe(SF)]	DCM	r.t.	6	n.d.
9	CyCHO	[Fe(SF)]	THF	80	6	n.d.
10[b]	CyCHO	[Fe(SF)]	ACN	80	3	65

Reactions were performed under the following conditions: styrene (1 mmol), co-reductant (1.5 mmol), 2 mL of solvent, 25 mg of catalyst, under oxygen atmosphere. Yields determined by GC-FID. [a] Reactions performed in atmospheric conditions [b] The catalyst was prepared using $FeCl_3$ as iron source

Table S2: Metal-based catalysts reported for the epoxidation reaction

**Catalyst**	**Substrate**	**Oxidant**	**Solvent**	**Metal Loading**	**Conversion (%)**	**References**
TS-1	Propylene	H_2O_2	Methanol / ACN	2 wt%	60–85	Snamprogetti S.p.A. [4-6]
Ag/Al_2O_3	Ethylene	O_2	None, vapor-phase process	1–25 wt%	80-99	Shell[7], DOW[8], BASF[9], SINOPEC[10]
Fe-porphyrin complex	Styrene	Fe(TPP)Cl + PhIO	Toluene	1–2 mol%	58-76	[11]
Hydrotalcite $Mg_{10}Al_2(OH)_{24}CO_3$	Cyclooctene	H_2O_2	1,2-dichloroethane	//	85-99	[12]
Peroxotungstates	Styrene, Octene	H_2O_2	1,2-dichloroethane	//	80-95%	[13]

Synthesis of 2-phenyloxirane (2a)[14]

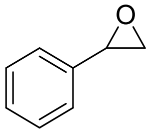

Compound 2a was obtained following the general procedure. Isolated yield 90% (108.1 mg).

^{1}H NMR (500 MHz, $CDCl_3$) δ 7.40 – 7.24 (m, 5H), 3.87 (dd, *J* = 4.2, 2.6 Hz, 1H), 3.15 (dd, *J* = 5.5, 3.8 Hz, 1H), 2.81 (dd, *J* = 5.5, 2.6 Hz, 1H).

^{13}C NMR (125 MHz, $CDCl_3$) δ 137.70, 128.60, 128.27, 125.58, 52.44, 51.27.

Synthesis of 2-(p-tolyl)oxirane (2b)[15]

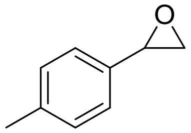

Compound 2b was obtained following the general procedure. Isolated yield 89% (119.4 mg).

^{1}H NMR (500 MHz, $CDCl_3$) δ 7.20 – 7.12 (m, 4H), 3.83 (dd, *J* = 4.1, 2.6 Hz, 1H), 3.13 (dd, *J* = 5.5, 4.1 Hz, 1H), 2.80 (dd, *J* = 5.5, 2.6 Hz, 1H), 2.35 (s, 3H).

^{13}C NMR (125 MHz, $CDCl_3$) δ 138.08, 134.63, 129.30, 125.58, 52.43, 51.19, 21.28.

Synthesis of 2-(4-chlorophenyl)oxirane (2c)[16]

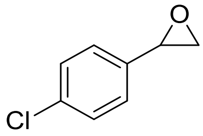

Compound 2c was obtained following the general procedure. Isolated yield 90% (139.1 mg).

^{1}H NMR (500 MHz, $CDCl_3$ δ 7.35 – 7.29 (m, 2H), 7.24 – 7.18 (m, 2H), 3.84 (dd, *J* = 4.1, 2.6 Hz, 1H), 3.15 (dd, *J* = 5.3, 4.0 Hz, 1H), 2.75 (dd, *J* = 5.5, 2.6 Hz, 1H).

^{13}C NMR (125 MHz, $CDCl_3$) δ 136.33, 134.11, 128.87, 126.99, 51.94, 51.39.

Synthesis of 4-(oxiran-2-yl)phenyl acetate (2d)[14]

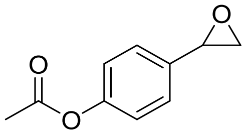

Compound 2d was obtained following the general procedure. Isolated yield 70% (124.7 mg).

^{1}H NMR (500 MHz, $CDCl_3$) δ 7.29 (d, *J* = 8.6 Hz, 2H), 7.07 (d, *J* = 8.5 Hz, 2H), 3.86 (dd, *J* = 3.9, 2.5 Hz, 1H), 3.14 (dd, *J* = 5.4, 4.0 Hz, 1H), 2.77 (dd, *J* = 5.5, 2.5 Hz, 1H), 2,30 (s, 3H).

^{13}C NMR (125 MHz, $CDCl_3$) δ 169.58, 150.69, 135.37, 126.71, 121.87, 52.06, 51.37, 21.25.

Synthesis of 2-methyl-3-phenyloxirane (2e)[14]

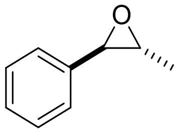

Compound 2e was obtained following the general procedure. Isolated yield 92% (123.4 mg).

^{1}H NMR (500 MHz, $CDCl_3$) δ 7.60 – 7.04 (m, 5H), 3.58 (d, *J* = 2.0 Hz, 1H), 3.11 – 2.75 (m, 1H), 1.46 (d, *J* = 5.2 Hz, 3H).

^{13}C NMR (125 MHz, $CDCl_3$) δ 137.88, 128.54, 128.14, 125.66, 59.64, 59.12, 18.01.

Synthesis of 9-oxabicyclo[6.1.0]nonane (2f)[17]

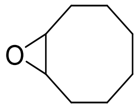

Compound 2f was obtained following the general procedure. Isolated yield 97% (122.4 mg).

^{1}H NMR (500 MHz, $CDCl_3$) δ 2.88 (d, *J* = 10.0 Hz, 2H), 2.13 (dd, *J* = 13.8, 4.1 Hz, 2H), 1.83 – 1.33 (m, 8H), 1.33 – 1.16 (m, 2H).

^{13}C NMR (125 MHz, $CDCl_3$) δ 55.76, 26.40, 25.71

Synthesis of *cis*-2,3-diphenyloxirane (2g)[18]

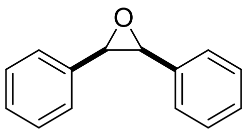

Compound 2g was obtained following the general procedure. Isolated yield 15% (29.4 mg).

^{1}H NMR (500 MHz, $CDCl_3$) δ 7.09–7.21 (m, 10H), 4.31 (s, 2H).

^{13}C NMR (125 MHz, $CDCl_3$) δ 134.48, 127.92, 127.65, 126.99, 59.91.

Synthesis of *trans*-2,3-diphenyloxirane (2h)[19]

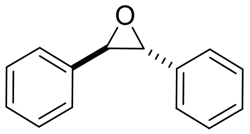

Compound 2h was obtained following the general procedure. Isolated yield 99% (194.2 mg).

^{1}H NMR (500 MHz, $CDCl_3$) δ 7.43-7.31 (m, 10H), 3.88 (s, 2H).

^{13}C NMR (125 MHz, $CDCl_3$) δ 137.29, 137.25, 128.73, 128.70, 128.49, 128.46, 125.68, 125.64, 62.97.

Synthesis of 2-(4-bromophenyl)-3-phenyloxirane (2i)[20]

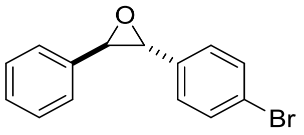

Compound 2i was obtained following the general procedure. Isolated yield 98% (296.6 mg).

^{1}H NMR (500 MHz, $CDCl_3$) δ 7.51 (d, *J* = 8.2 Hz, 2H), 7.44 – 7.30 (m, 5H), 7.23 (d, *J* = 8.3 Hz, 2H), 3.90 – 3.70 (m, 2H).

^{13}C NMR (125 MHz, $CDCl_3$) δ 136.83, 136.31, 131.84, 128.75, 128.62, 127.29, 125.62, 122.34, 63.00, 62.33.

Synthesis of 2-(4-nitrophenyl)-3-phenyloxirane (2j)[21]

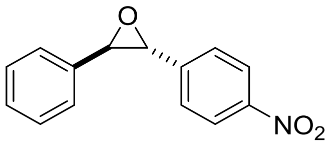

Compound 2*J* was obtained following the general procedure. Isolated yield 82% (197.8 mg).

^{1}H NMR (500 MHz, $CDCl_3$) δ 8.24 (d, *J* = 8.3 Hz, 2H), 7.51 (d, *J* = 8.1 Hz, 2H), 7.38 (dt, *J* = 20.1, 7.2 Hz, 5H), 3.98 (s, 1H), 3.86 (s, 1H).

^{13}C NMR (125 MHz, $CDCl_3$) δ 147.96, 144.55, 136.19, 128.93, 128.84, 126.38, 126.35, 125.66, 124.00, 63.46, 61.76.

Synthesis of 4-(-3-phenyloxiran-2-yl)benzaldehyde (2k)

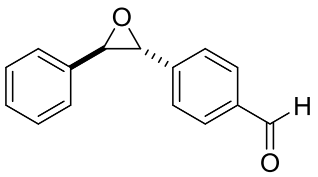

Compound 2k was obtained following the general procedure. Isolated yield 97% (217.5 mg).

^{1}H NMR (500 MHz, $CDCl_3$) δ 10.03 (s, 1H), 7.90 (d, *J* = 7.9 Hz, 2H), 7.52 (d, *J* = 7.8 Hz, 2H), 7.46 – 7.32 (m, 5H), 3.95 (d, *J* = 1.7 Hz, 1H), 3.87 (d, *J* = 1.8 Hz, 1H).

^{13}C NMR (125 MHz, $CDCl_3$) δ 191.86, 144.08, 136.56, 136.47, 130.16, 128.82, 128.81, 126.19, 125.69, 63.34, 62.32.

Synthesis of ethyl 4-(3-phenyloxiran-2-yl)benzoate (2l)

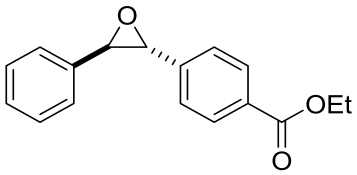

Compound 2l was obtained following the general procedure. Isolated yield 55% (147.5 mg).

^{1}H NMR (500 MHz, $CDCl_3$) δ 8.07 (d, *J* = 7.9 Hz, 2H), 7.39 (dt, *J* = 22.8, 7.7 Hz, 7H), 4.39 (q, *J* = 7.1 Hz, 2H), 3.92 (s, 1H), 3.87 (s, 1H), 1.41 (t, *J* = 7.1 Hz, 3H).

^{13}C NMR (125 MHz, $CDCl_3$) δ 166.27, 142.13, 130.46, 129.87, 128.66, 128.58, 125.59, 125.58, 125.45, 63.10, 62.34, 61.08, 14.38.

Synthesis of phenyl 3-phenyloxiran-2-yl)methanone (2m)[22]

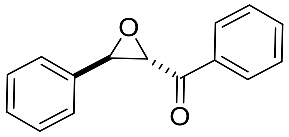

Compound 2m was obtained following the general procedure. Isolated yield 35% (78.5 mg).

^{1}H NMR (500 MHz, $CDCl_3$) δ 8.01 (dd, *J* = 7.6, 1.6 Hz, 2H), 7.69 – 7.58 (m, 1H), 7.49 (t, *J* = 7.7 Hz, 2H), 7.39 (m, 5H), 4.30 (d, *J* = 2.0 Hz, 1H), 4.08 (d, *J* = 1.9 Hz, 1H).

^{13}C NMR (125 MHz, $CDCl_3$) δ 193.25, 135.64, 134.15, 129.22, 129.04, 128.94, 128.52, 125.95, 61.19, 59.54.

Synthesis of ethyl 2-cyano-3-phenyloxirane-2-carboxylate (2n)[22]

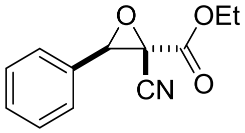

Compound 2n was obtained following the general procedure. Isolated yield 40% (86.9 mg).

^{1}H NMR (500 MHz, $CDCl_3$) δ 7.45 (m, 5H), 4.52 (d, *J* = 2.7 Hz, 1H), 4.41 (m, 2H), 1.40 (t, *J* = 7.1 Hz, 3H).

^{13}C NMR (125 MHz, $CDCl_3$) δ 162.82, 130.53, 129.92, 128.92, 126.88, 113.05, 64.57, 64.28, 53.44, 14.11.

Synthesis of cholesterol-5,6-epoxide (2o)[23-24]

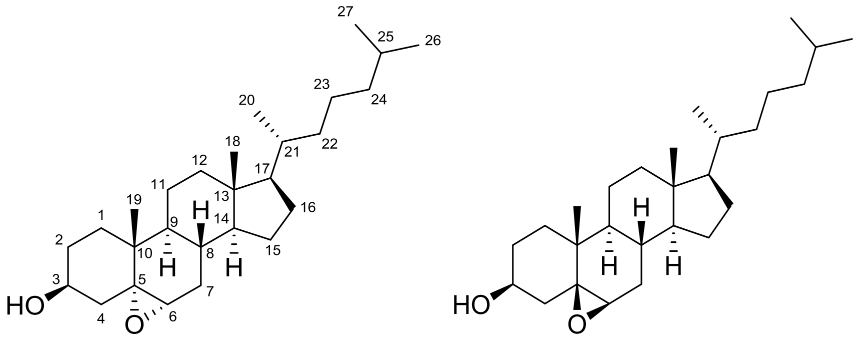

Cholesterol-5α,6α-epoxide Cholesterol-5β,6β-epoxide

Compound 2o was obtained following the general procedure. Isolated yield of 96% (386.5 mg) of a mixture of cholesterol α- and β-epoxide, with a ratio of 1.0:1.9.

^{1}H NMR (500 MHz, $CDCl_3$): δ 3.89 (m, 1H, H-3 α-epoxide), 3.68 (m, 1H, H-3 β -epoxide), 3.05 (s, 1H, H-6 β-epoxide), 2.89 (d, *J* = 4.4 Hz, 1H, H-6 α-epoxide) 0.63 (s, 3H, H-18 β-epoxide), 0.60 (s, 3H, H-18 α-epoxide).

^{13}C NMR (125 MHz, $CDCl_3$): δ 69.50(C-5), 68.79(C-3), 65.89(C-5), 63.87, 63.11, 59.45(C-6), 56.97(C-17), 56.34, 56.32, 55.98(C-14), 51.45, 42.68(C-9), 42.45(C-13), 42.40, 42.33, 39.95(C-12), 39.61(C-24), 39.53(C-4), 37.35, 36.25(C-22), 35.84(C-20), 34.97(C-10), 32.72, 32.52(C-1), 31.17(C-7), 31.13(C-7), 30.00(C-8), 29.89(C-2), 28.93(C-2), 28.27(C-16), 28.12(C-25), 24.31(C-15), 23.96(C-23), 23.93(C-23), 22.94(C-27), 22.92(C-27), 22.67(C-26), 22.11, 20.76(C-11), 18.79(C-21), 18.76(C-21), 17.17, 16.04(C-19), 11.98(C-18), 11.88 (C-18).

Synthesis of limonene epoxide[25-26]

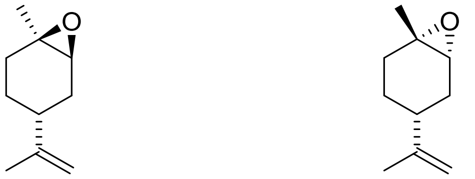

cis-limonene oxide *trans*-limonene oxide

Compound 2p was obtained following the general procedure. Isolated yield of 70% (106 mg) of a mixture of *cis*- and *trans*-limonene epoxide, with a ratio of 1.2.

^{1}H NMR (500 MHz, $CDCl_3$): δ 4.72-4.67 (m, 2H), 3.05 (t, *J* = 2.2 Hz, *cis*-, 1H), 2.99 (d, *J* = 5.2 Hz, *trans*-, 1H), 2.16 – 1.99 (m, 2H), 1.87 – 1.82 (m, 1H), 1.70 – 1.65 (m, 5H), 1.39 – 1.17 (m, 5H).

^{13}C NMR (125 MHz, $CDCl_3$): 149.38, 149.17, 109.21, 109.16, 60.69, 59.43, 57.68, 57.51, 40.86, 36.32, 30.86, 30.83, 29.99, 28.74, 26.04, 24.45, 24.43, 23.24, 21.24.

Recycle tests of [Fe(SF)] in the epoxidation reaction of styrene (1a)

In a 25 mL round-bottom flask, styrene (1 mmol) and cy-CHO (1.5 mmol) were dissolved in ACN, (2.0 mL). [Fe(SF)] catalyst (25 mg) was then added to the solution. The reaction mixture was purged with oxygen and stirred at 80 °C for 3 h. After cooling to room temperature, the mixture was diluted with ACN and EtOAc and filtered through a Büchner funnel. The solid catalyst was thoroughly washed with ACN, EtOAc, and EtOH to remove any residual reagents or products. The filtrate was extracted with EtOAc, washed with saturated aqueous solution of $NaHCO_3$, dried over anhydrous Na_2SO_4, and the solvent was removed under reduced

pressure. The recovered catalyst was dried, weighed, and reused for subsequent epoxidation cycles. In each reuse, the amounts of reagents were adjusted according to the recovered mass of [Fe(SF)] to maintain a consistent catalyst-to-substrate ratio. Substrate conversion was determined by GC analysis. The recycling tests were carried out on two different batches of the catalyst, and the results reported in Figure 5 represent average values.

Hot filtration test of [Fe(SF)] in the epoxidation of styrene (1a)

In two 25 mL round-bottom flasks, styrene (1 mmol) and cy-CHO (1.5 mmol) were mixed into ACN (2.0 mL), then [Fe(SF)] catalyst (25 mg) was added. The reaction mixture was purged with oxygen and stirred at 80 °C for 2 h. In one case, the reaction mixture was cooled to room temperature, diluted with EtOAc, filtered through Buchner funnel, and filtrate was then extracted with EtOAc, washed saturated aqueous solution of $NaHCO_3$, dried over anhydrous Na_2SO_4 and the solvent was removed under vacuum, giving a 49% yield (determined by GC-MS analysis). In the other case, the reaction mixture was filtered at 80 °C on celite, and the clear solution obtained was introduced into another 25 mL round-bottom flask and stirred at 80 °C for further 1 h. After the usual work up, yield (49%) was determined by GC-MS analysis.

^{1}H-NMR and ^{13}C-NMR spectra

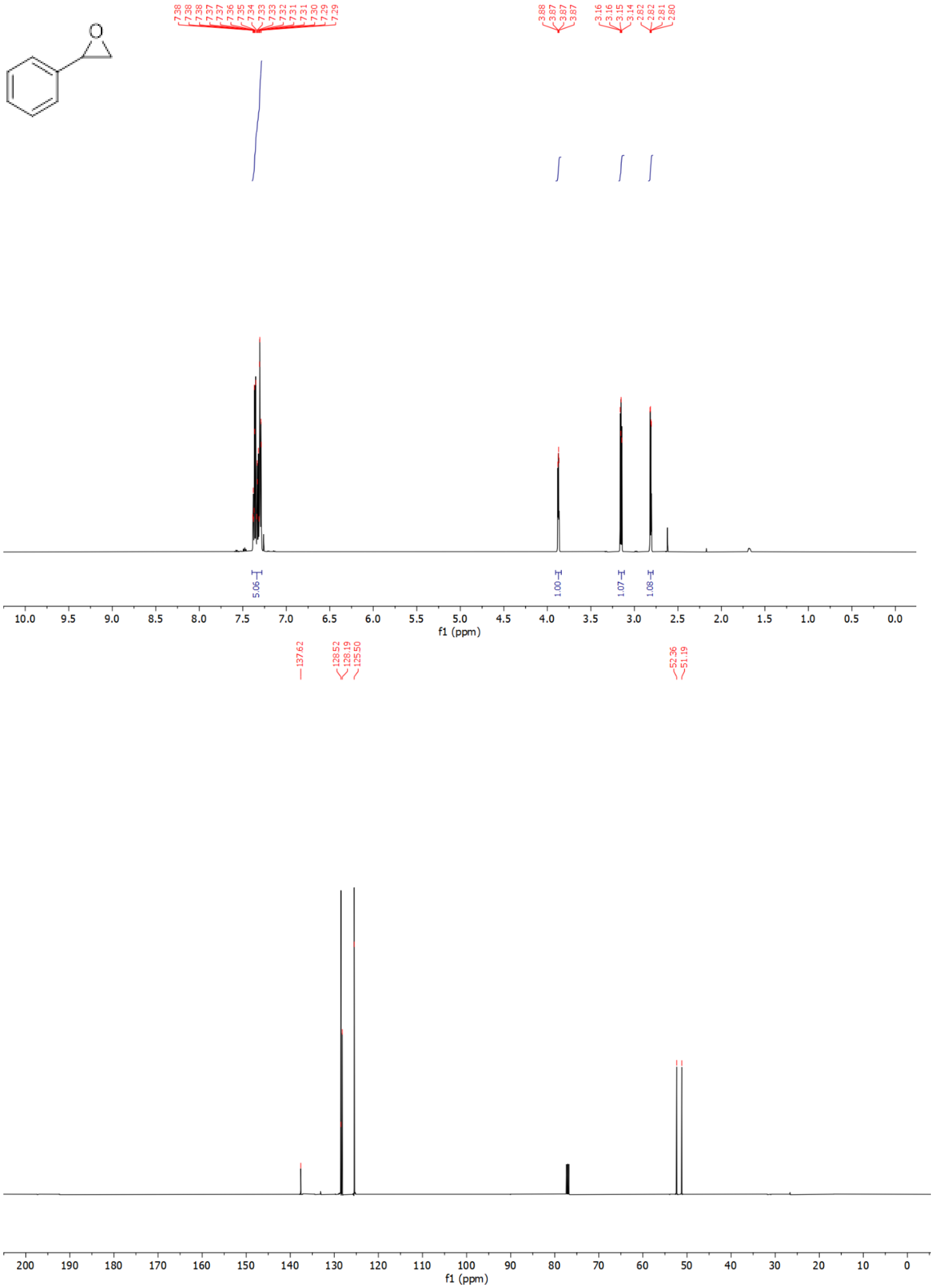

Figure S 7: ^{1}H NMR and ^{13}C NMR spectra of compound 2a (500 and 126 MHz, $CDCl_3$).

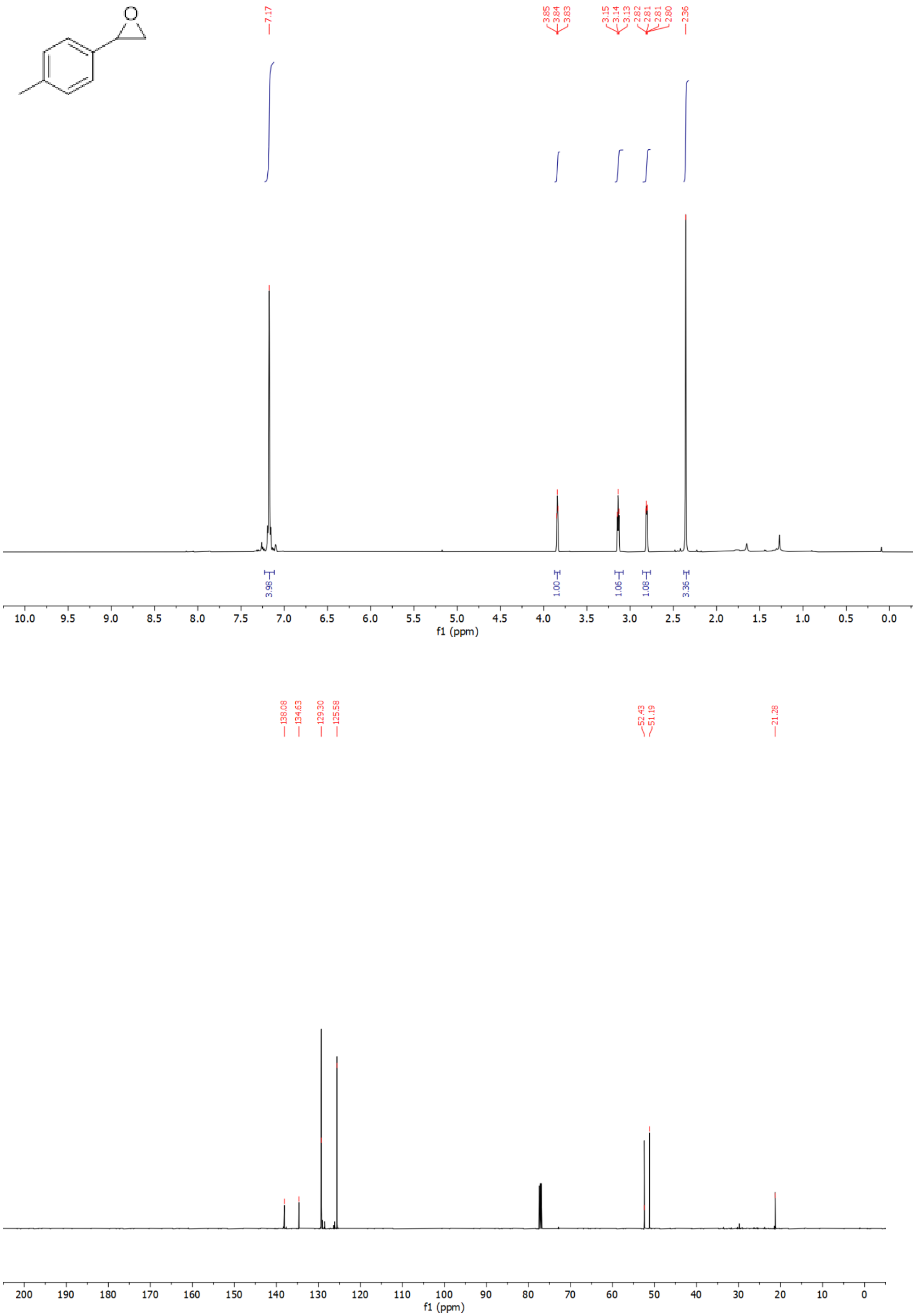

Figure S 8: ^{1}H NMR and ^{13}C NMR spectra of compound 2b (500 and 126 MHz, $CDCl_3$).

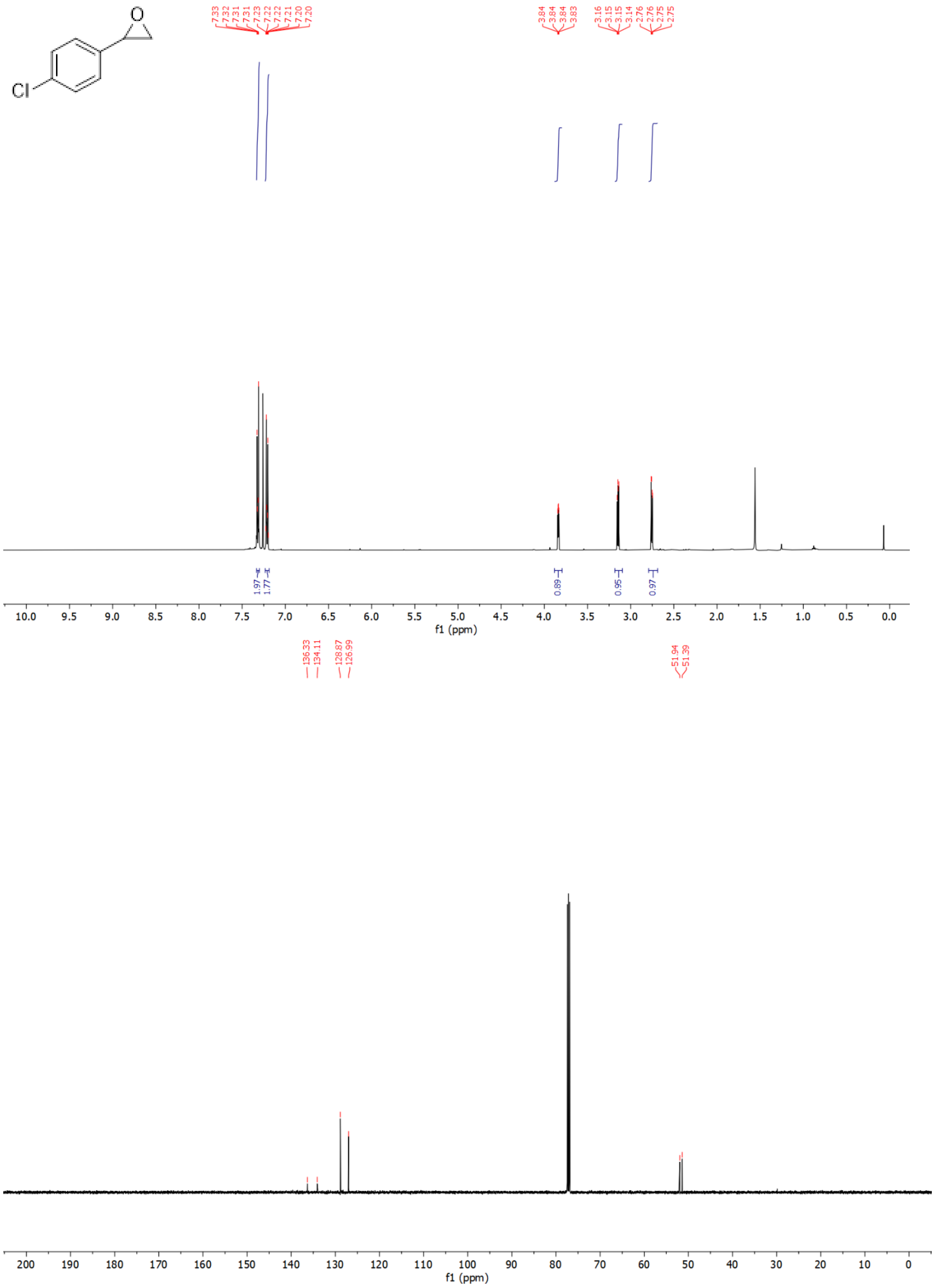

Figure S 9: ^{1}H NMR and ^{13}C NMR spectra of compound 2c (500 and 126 MHz, $CDCl_3$).

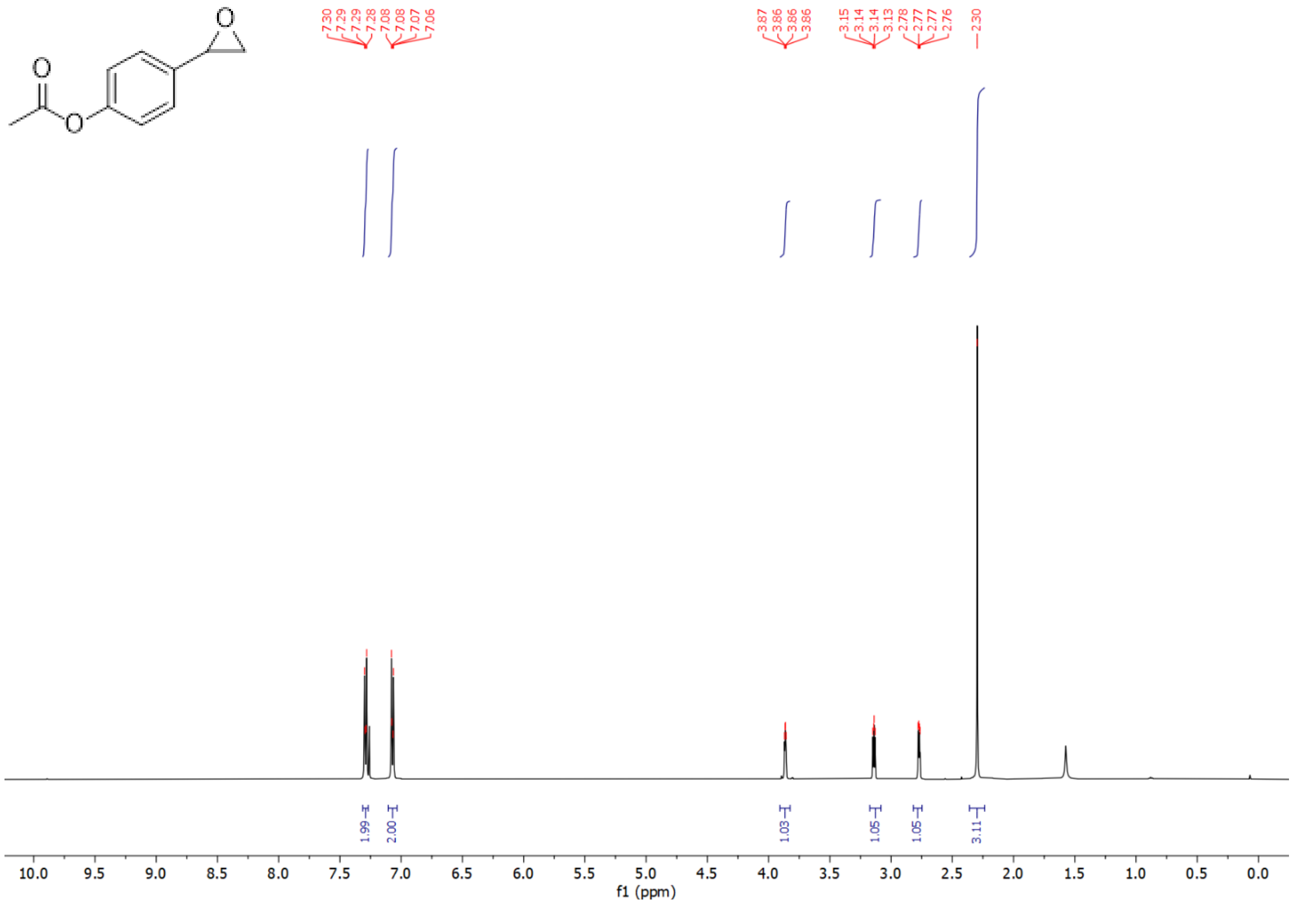

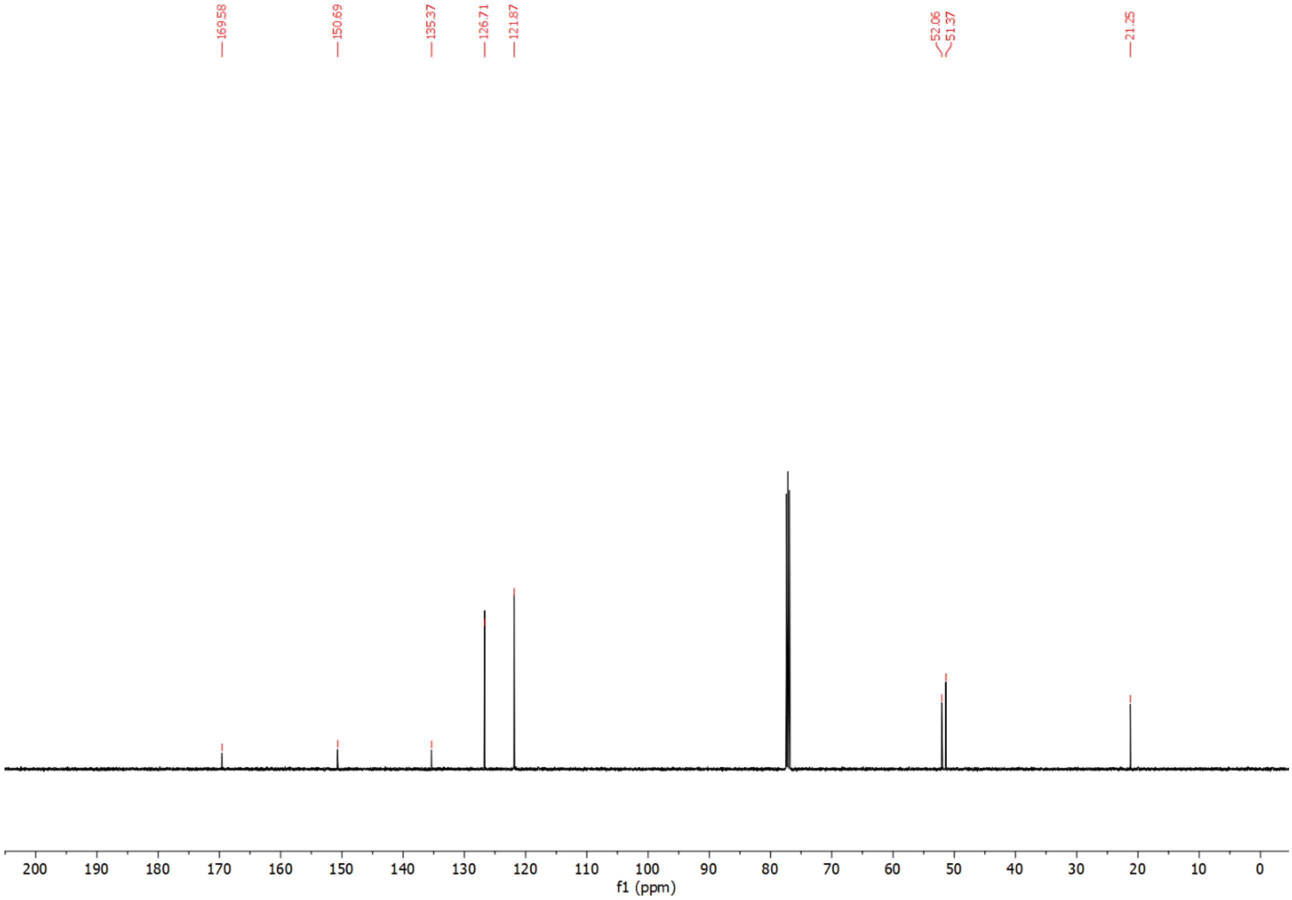

Figure S 10: ^{1}H NMR and ^{13}C NMR spectra of compound 2d (500 and 126 MHz, $CDCl_3$).

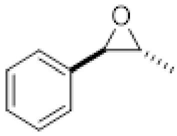

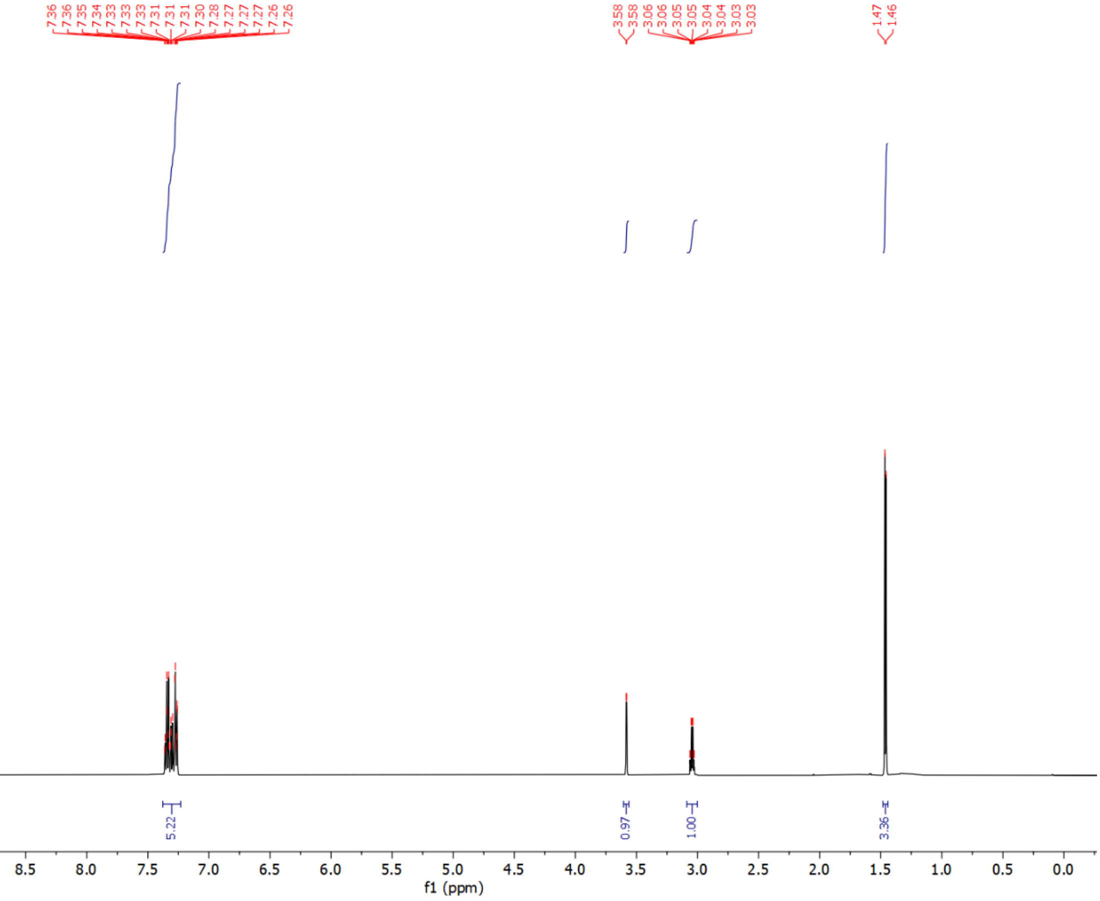

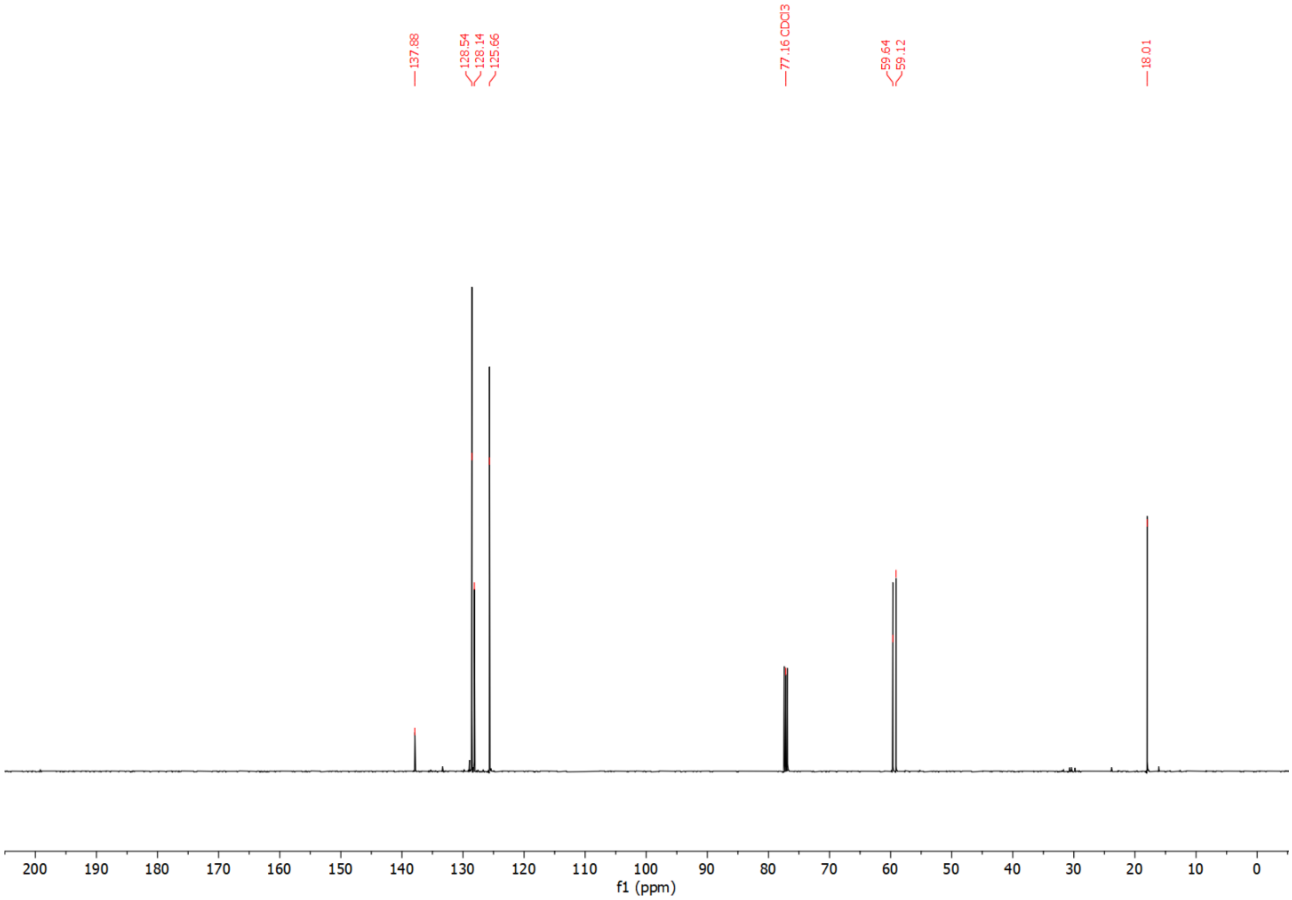

Figure S 11: ^{1}H NMR and ^{13}C NMR spectra of compound 2e (500 and 126 MHz, $CDCl_3$).

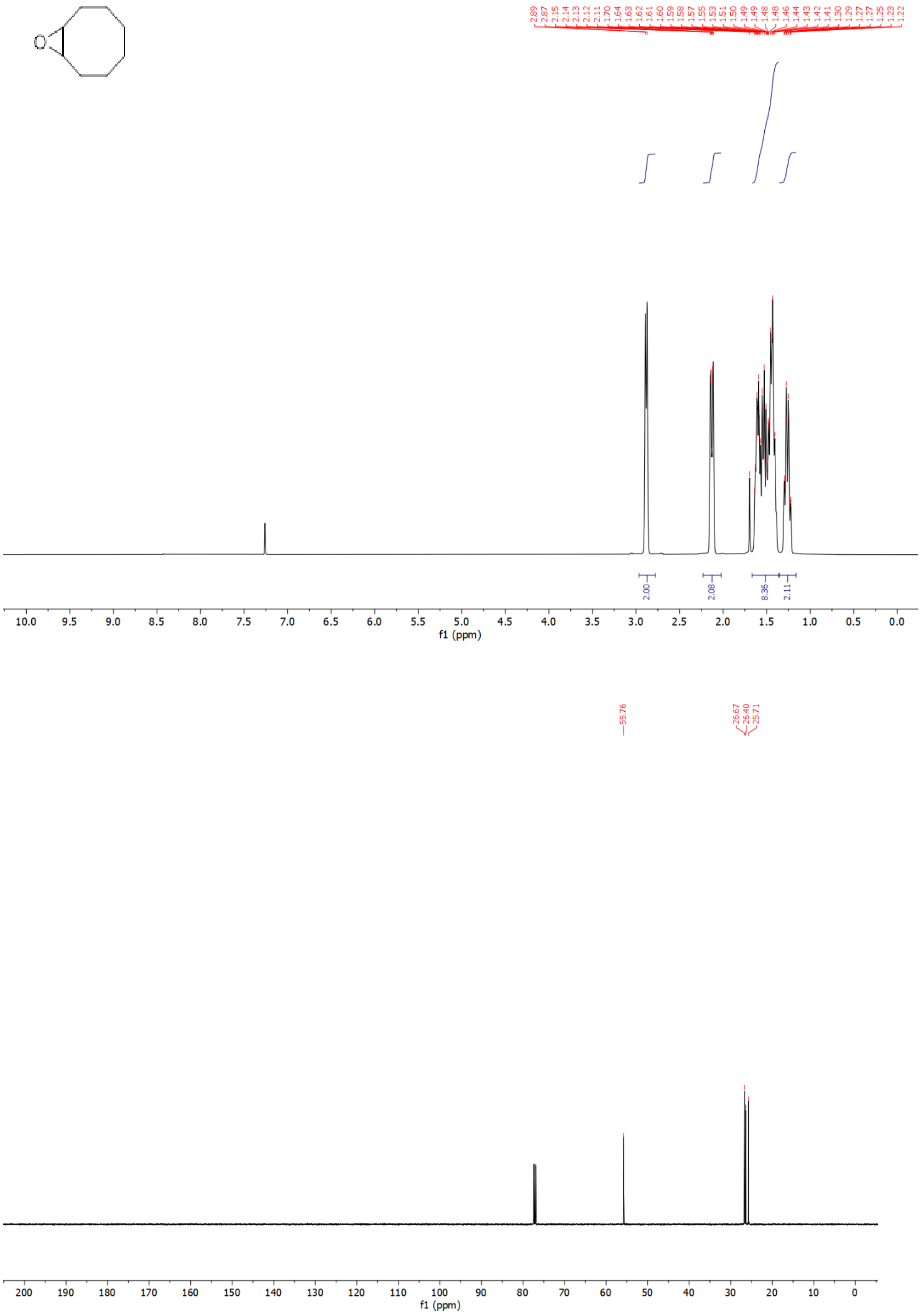

Figure S 12: ^{1}H NMR and ^{13}C NMR spectra of compound 2f (500 and 126 MHz, $CDCl_3$).

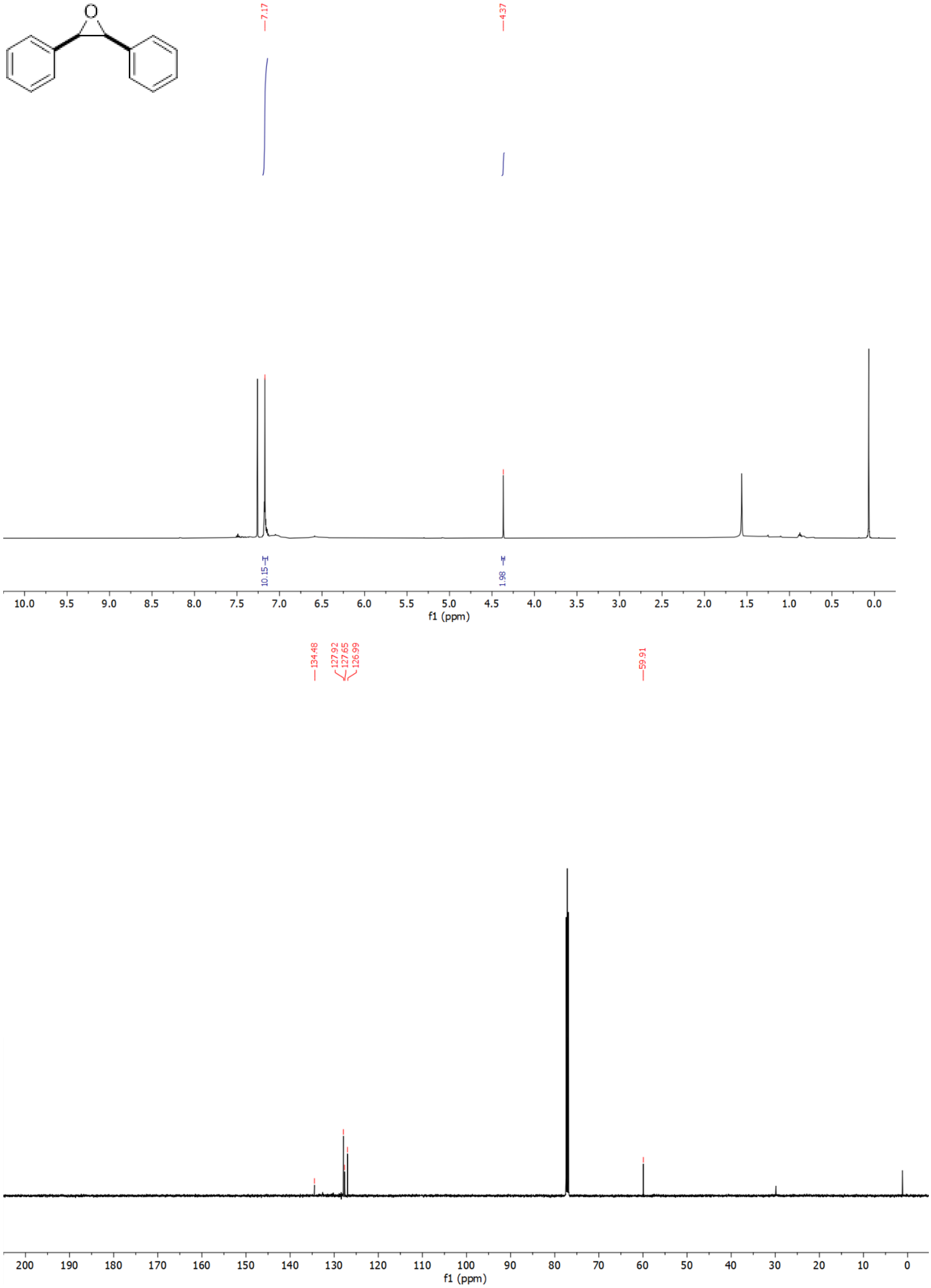

Figure S 13: ^{1}H NMR and ^{13}C NMR spectra of compound 2g (500 and 126 MHz, $CDCl_3$).

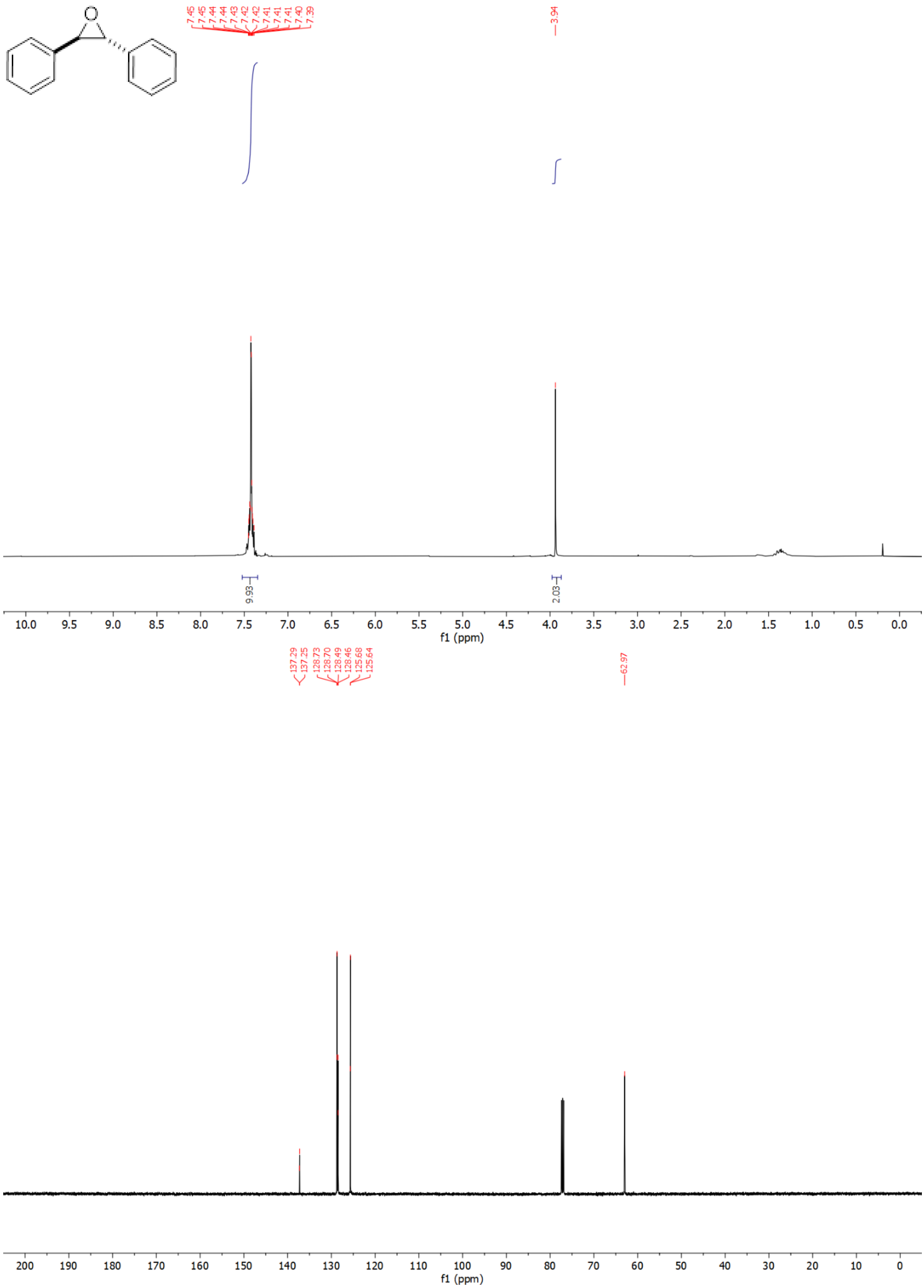

Figure S 14: ^{1}H NMR and ^{13}C NMR spectra of compound 2h (500 and 126 MHz, $CDCl_3$).

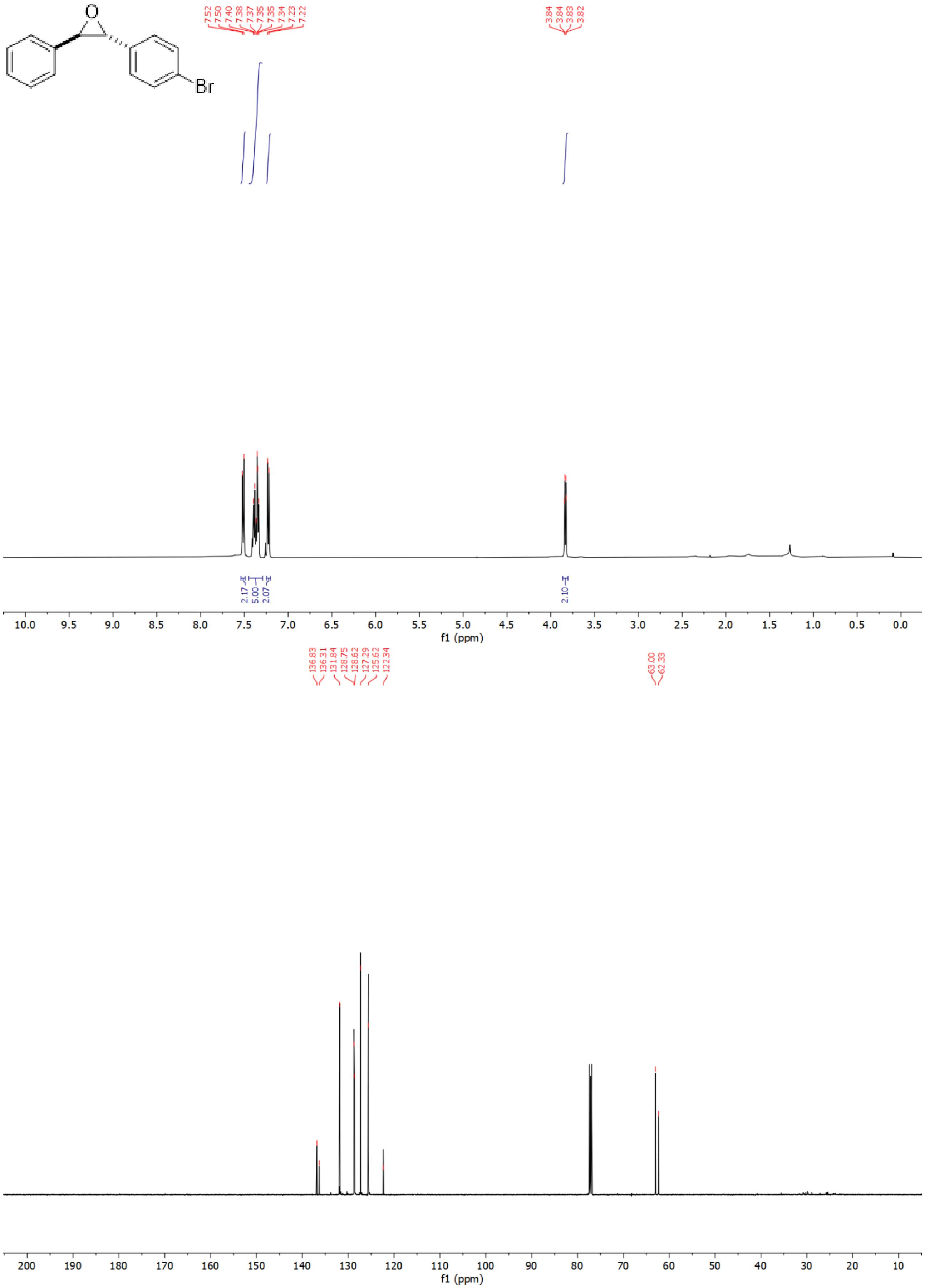

Figure S 15: ^{1}H NMR and ^{13}C NMR spectra of compound 2i (500 and 126 MHz, $CDCl_3$).

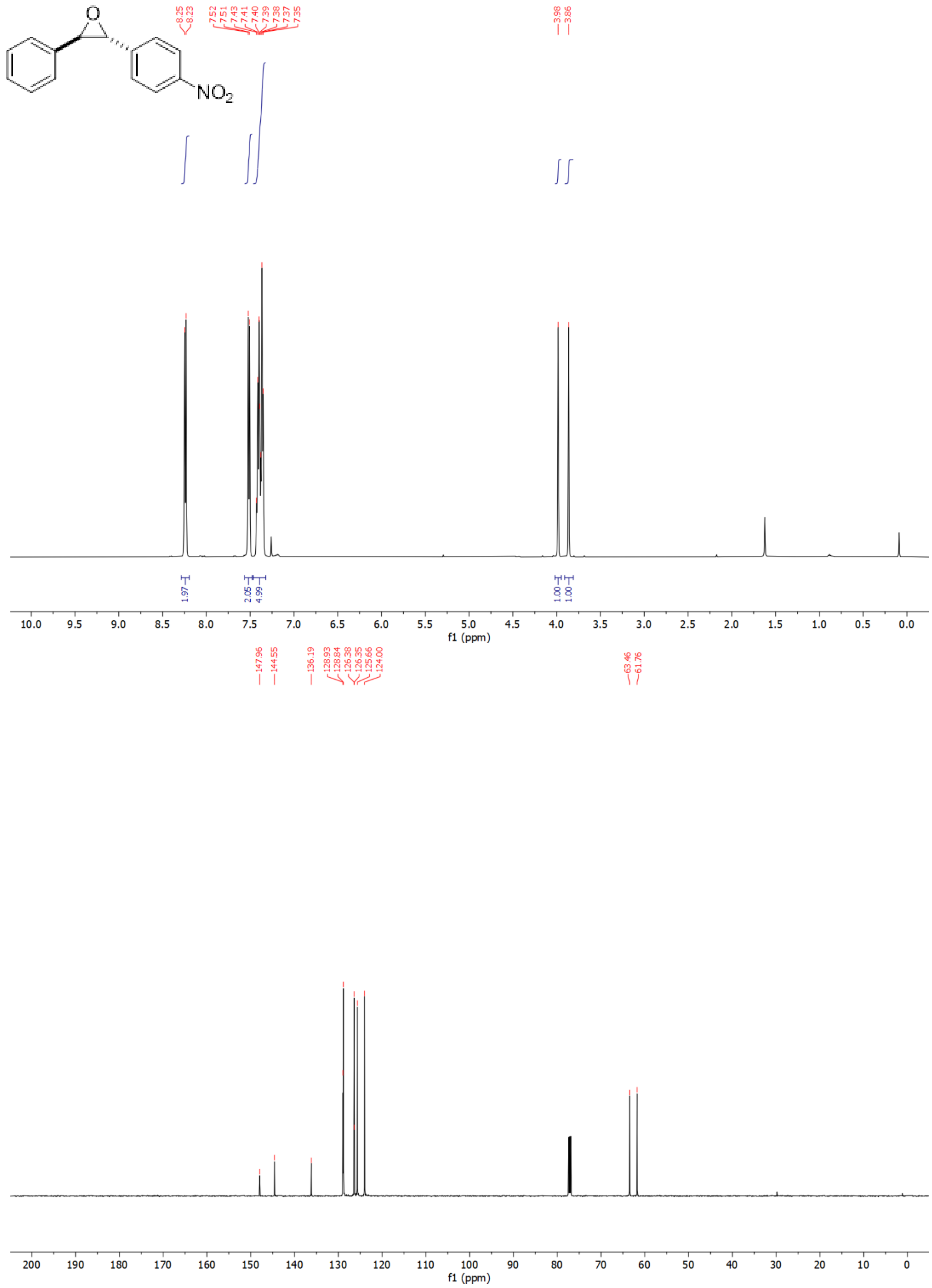

Figure S 16: ^{1}H NMR and ^{13}C NMR spectra of compound 2j (500 and 126 MHz, $CDCl_3$).

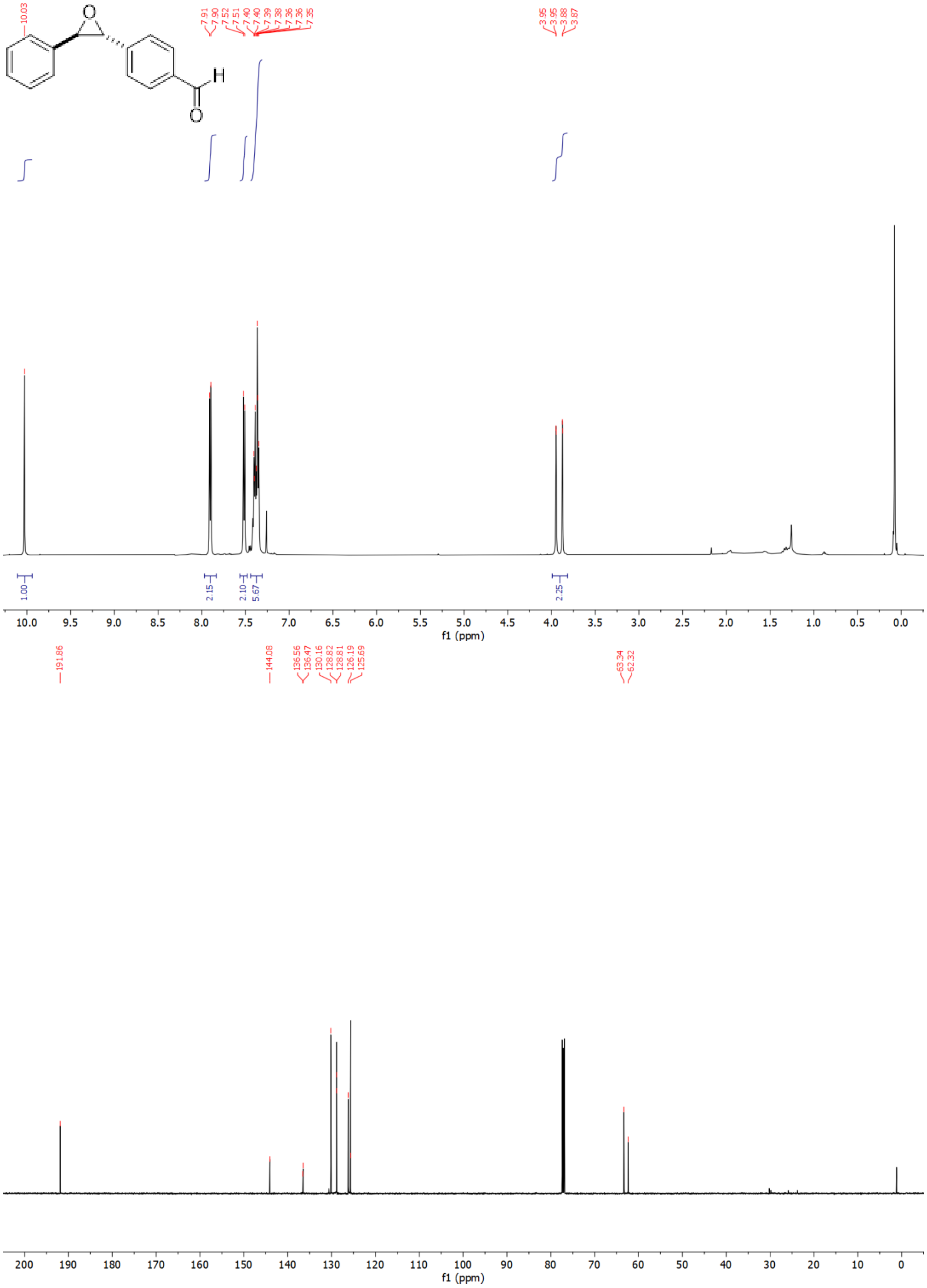

Figure S 17: ^{1}H NMR and ^{13}C NMR spectra of compound 2k (500 and 126 MHz, $CDCl_3$).

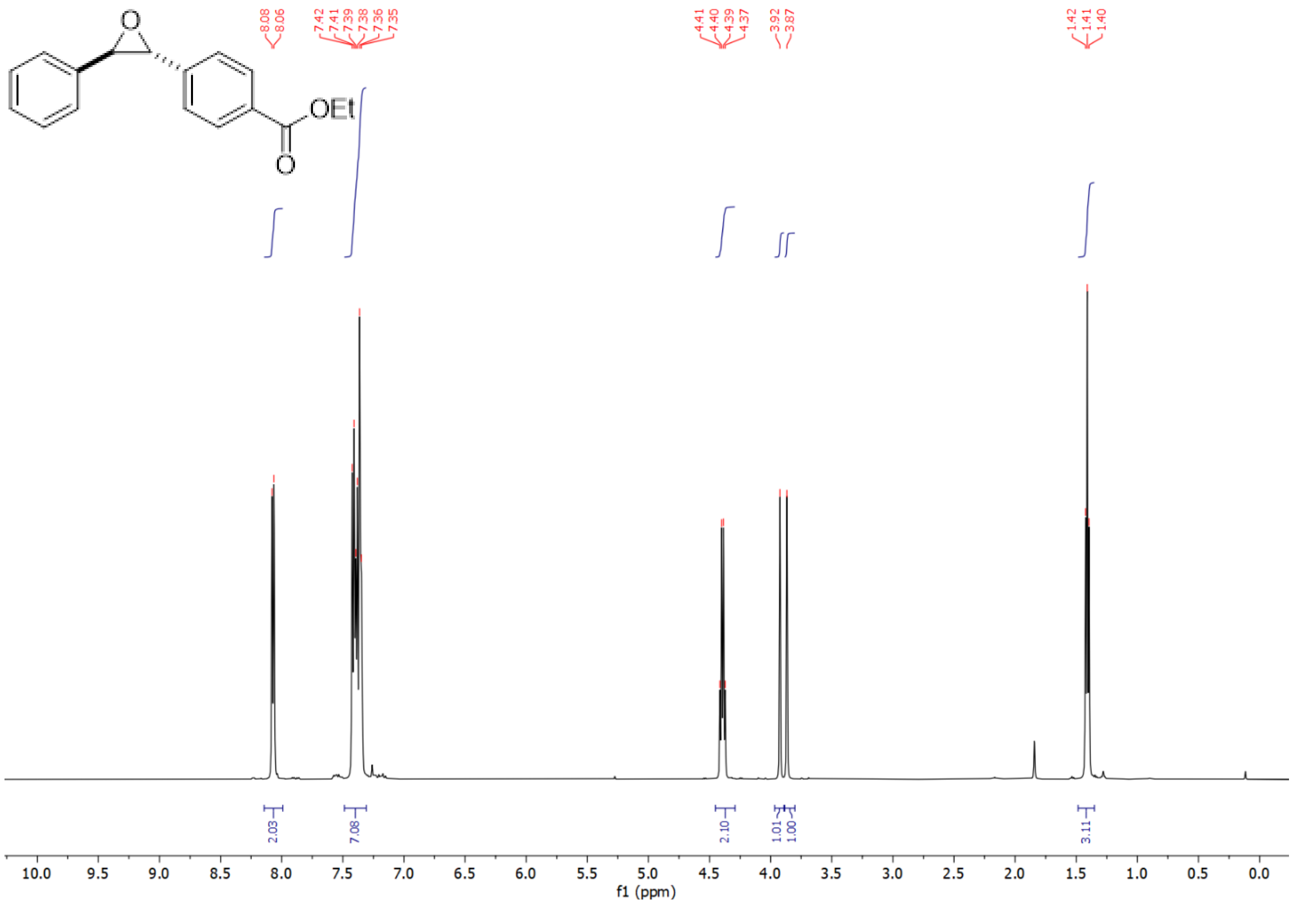

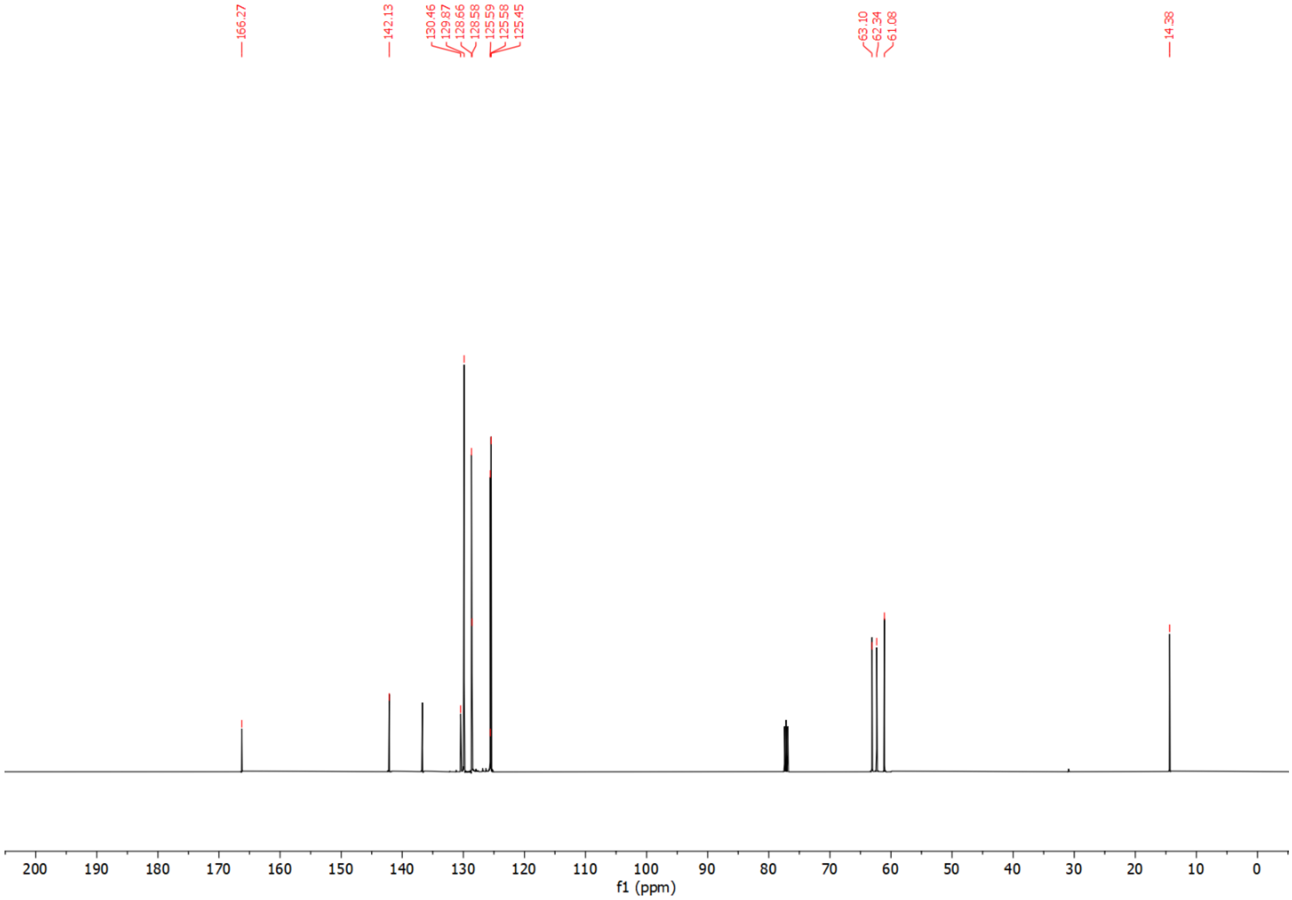

Figure S 18: ^{1}H NMR and ^{13}C NMR spectra of compound 2l (500 and 126 MHz, $CDCl_3$).

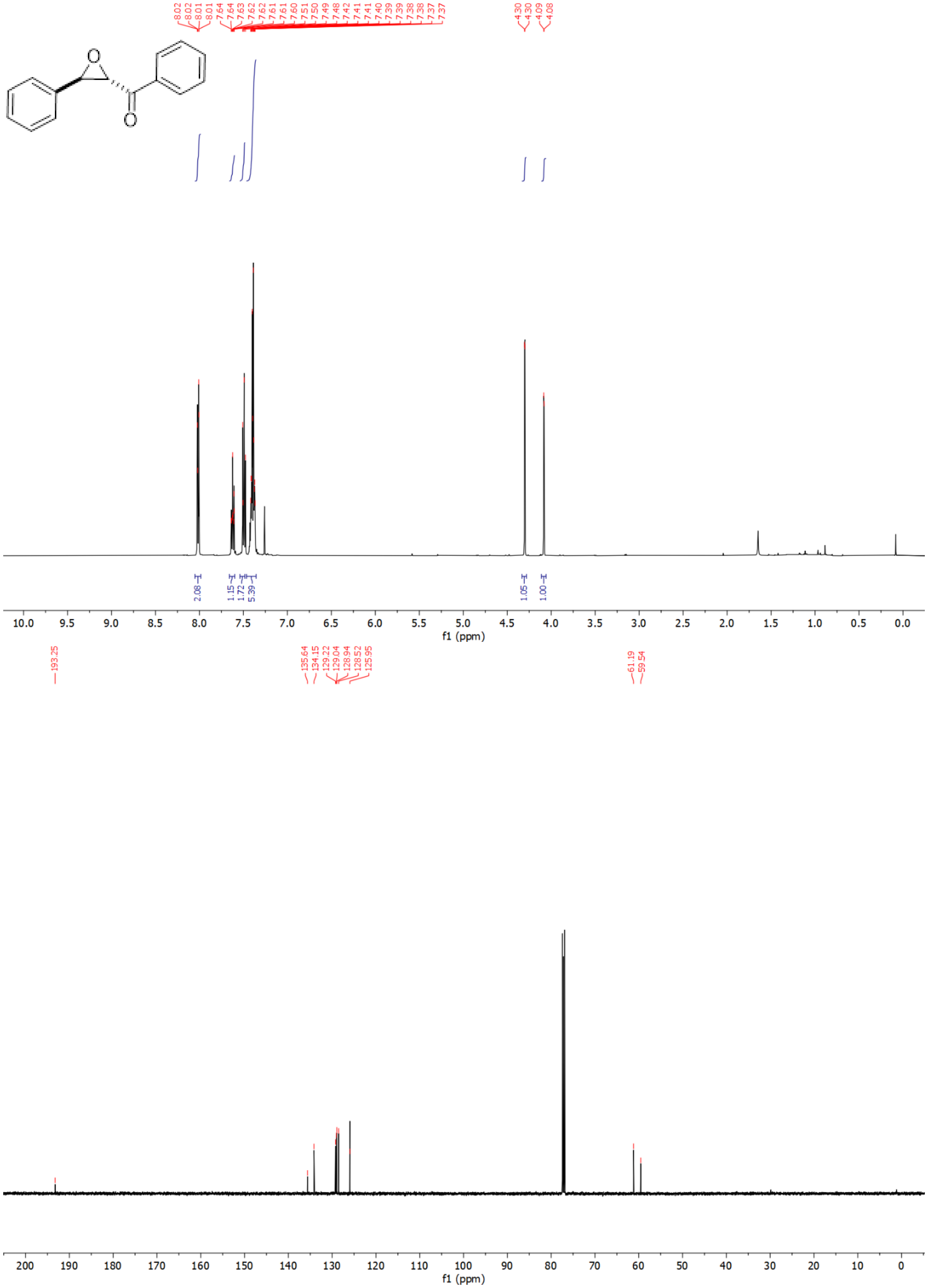

Figure S 19: ^{1}H NMR and ^{13}C NMR spectra of compound 2m (500 and 126 MHz, $CDCl_3$).

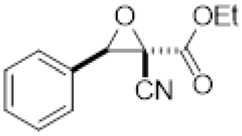

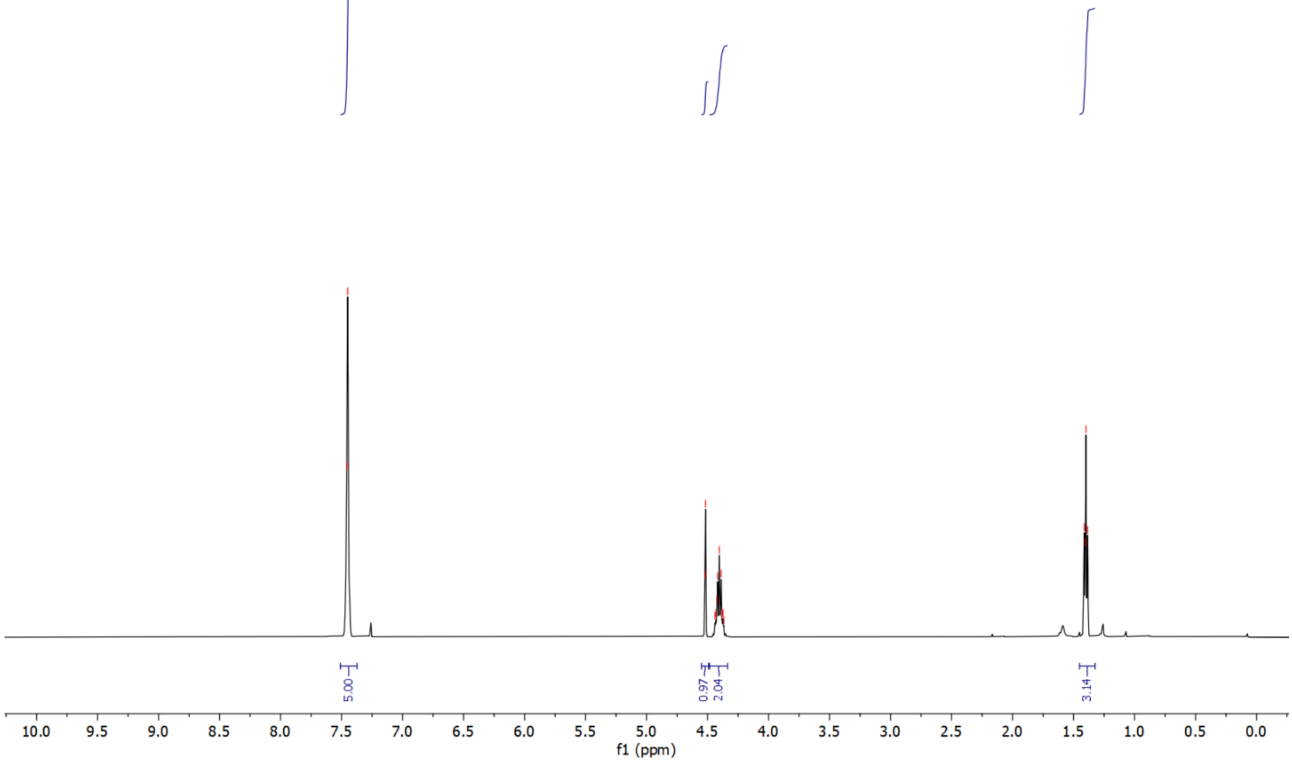

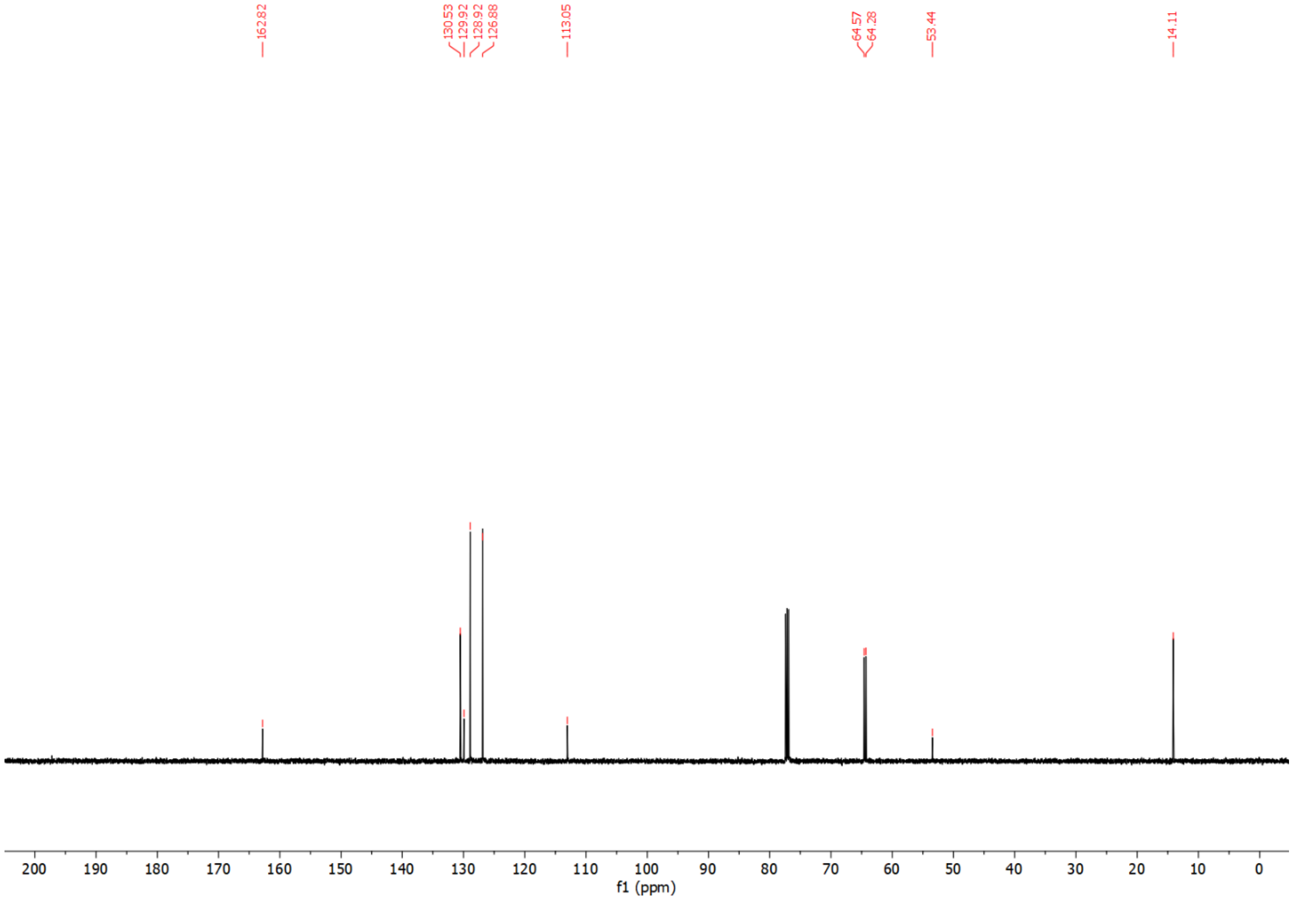

Figure S 20: ^{1}H NMR and ^{13}C NMR spectra of compound 2n (500 and 126 MHz, $CDCl_3$).

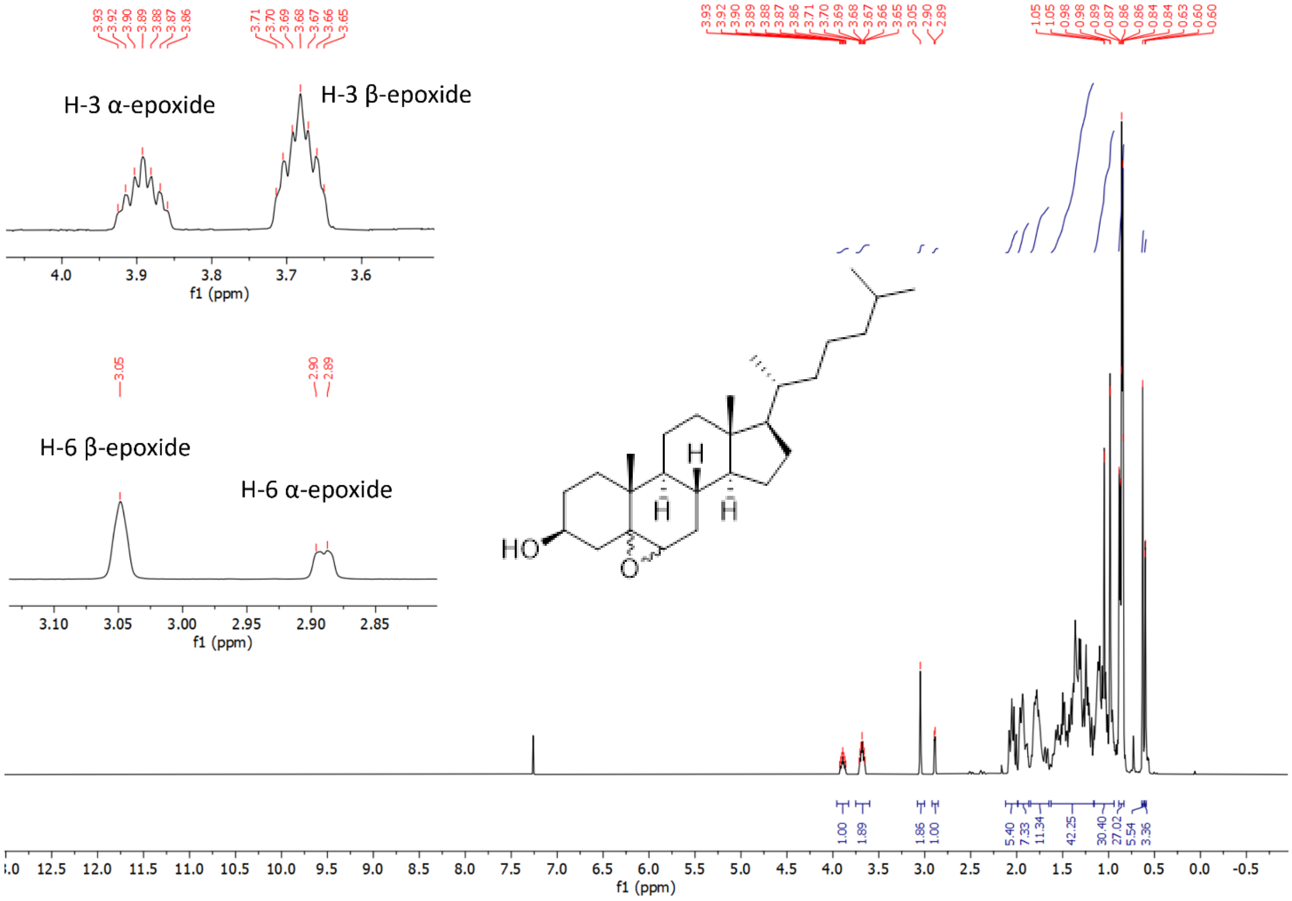

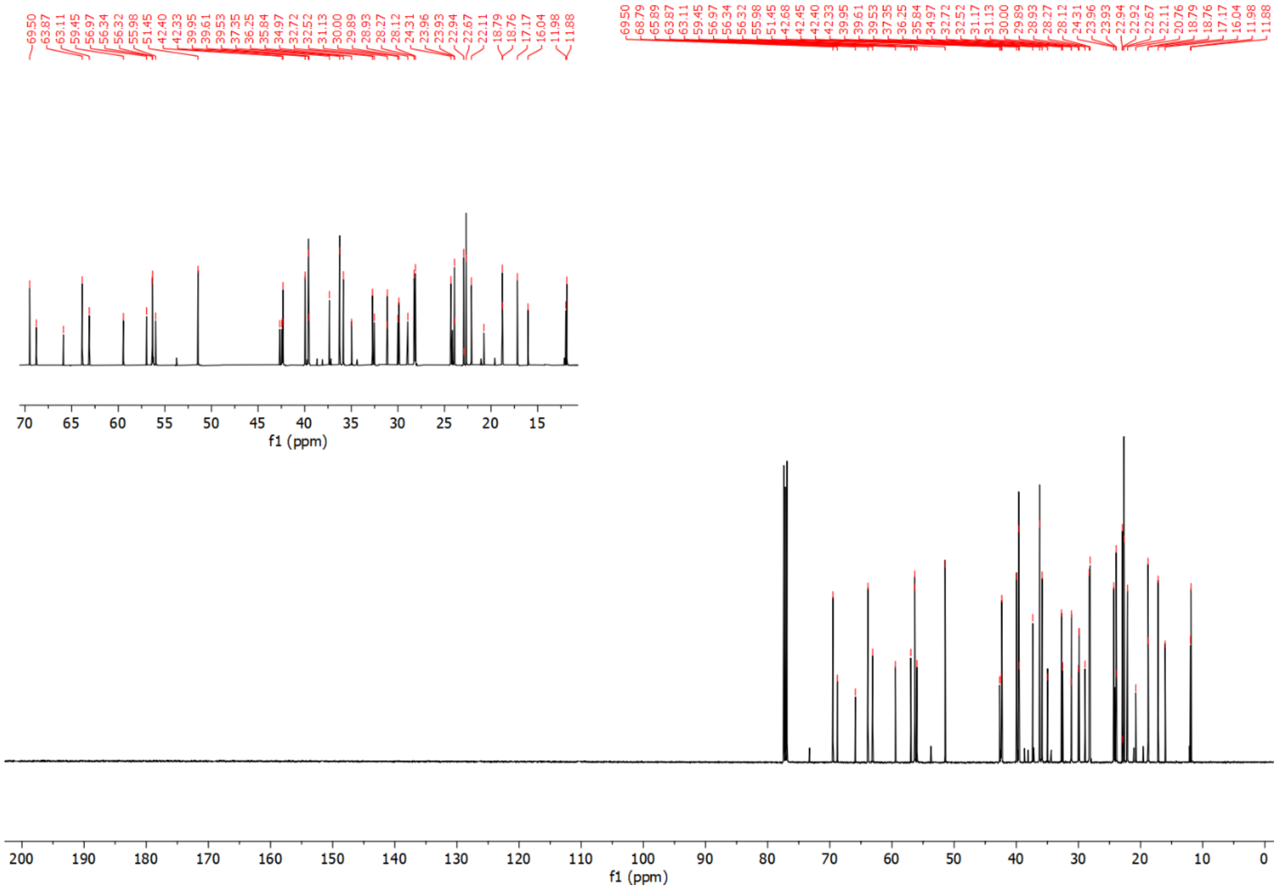

Figure S 21: ^{1}H NMR and ^{13}C NMR spectra of compound 2o (500 and 126 MHz, $CDCl_3$).

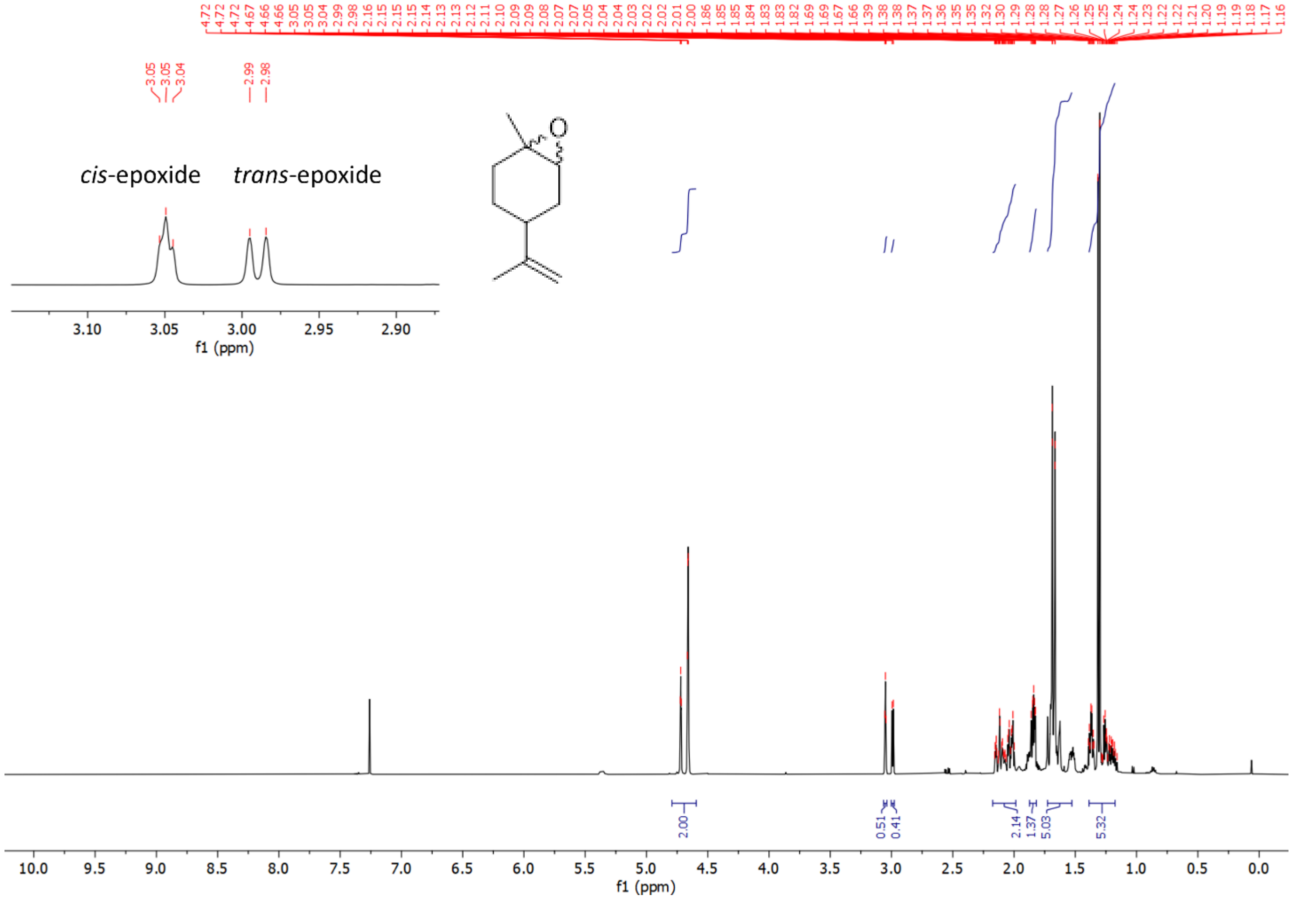

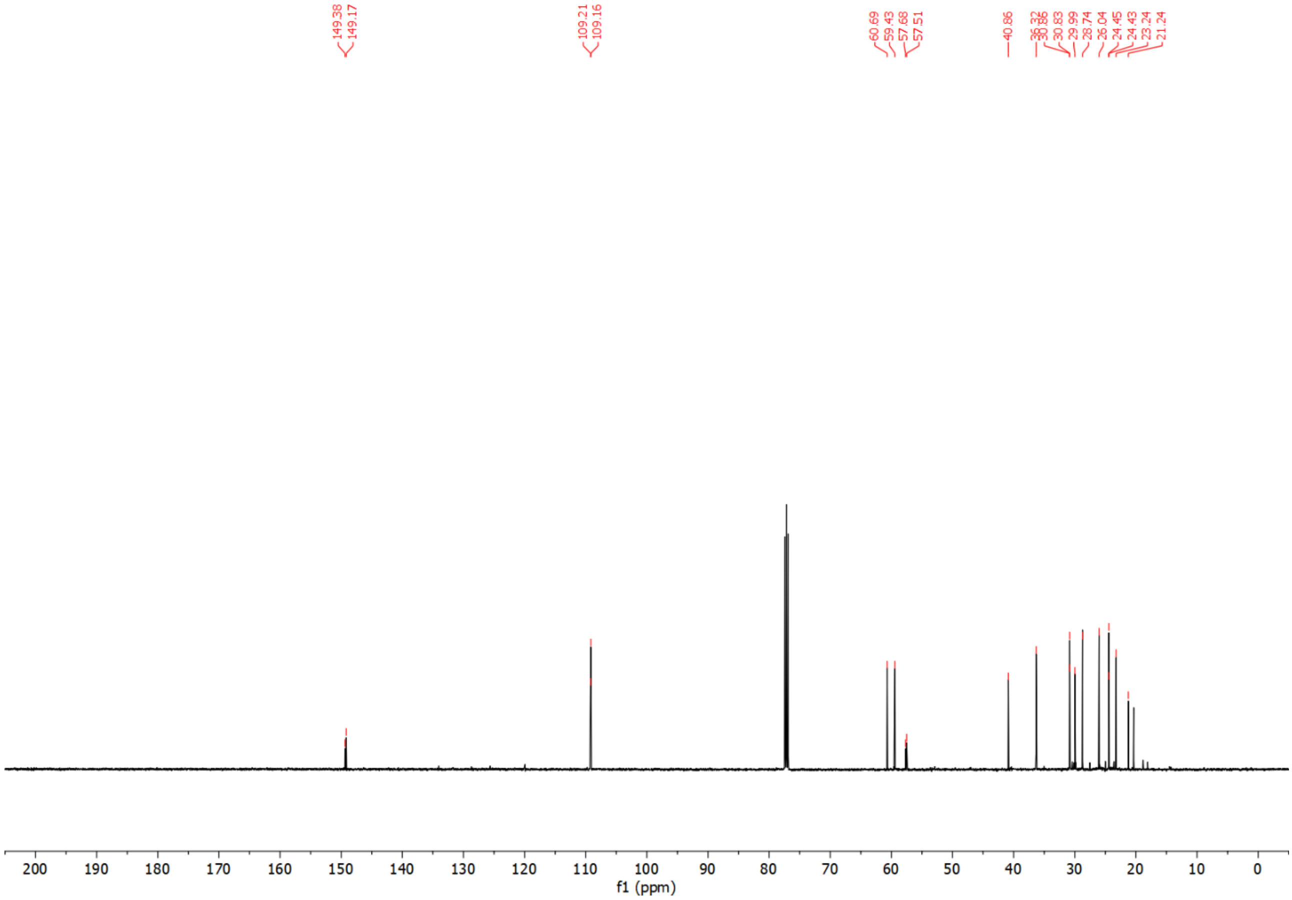

Figure S 22: ^{1}H NMR and ^{13}C NMR spectra of compound 2p (500 and 126 MHz, $CDCl_3$).

References

Uncategorized References

[1] D. N. Rockwood, R. C. Preda, T. Yücel, X. Wang, M. L. Lovett, D. L. Kaplan, *Nat. Protoc.* **2011**, *6*, 1612-1631.
[2] H. Dou, B. Zuo, *The Journal of The Textile Institute* **2015**, *106*, 311-319.
[3] I. C. Um, H. Kweon, Y. H. Park, S. Hudson, *International journal of biological macromolecules* **2001**, *29*, 91-97.
[4] M. Taramasso, G. Perego, B. Notari, Google Patents, **1983**.
[5] M. G. Clerici, G. Bellussi, U. Romano, *Journal of Catalysis* **1991**, *129*, 159-167.
[6] W. Zhang, J. L. Loebach, S. R. Wilson, E. N. Jacobsen, *Journal of the American Chemical Society* **1990**, *112*, 2801-2803.
[7] A. M. Lauritzen, Google Patents, **1988**.
[8] L. Zhang, W. H. Henstock, H. Soo, Google Patents, **2013**.
[9] A. Karpov, M. O. Kennema, N. Duyckaerts, C. Walsdorff, C. Bartosch, J. Zuehlke, M. A. R. Valle, Google Patents, **2024**.
[10] J. Chen, J. Jiang, S. Wang, Z. Zhang, J. Li, Google Patents, **2014**.
[11] J. T. Groves, R. S. Myers, *Journal of the American Chemical Society* **1983**, *105*, 5791-5796.
[12] K. Yamaguchi, K. Ebitani, K. Kaneda, *The Journal of Organic Chemistry* **1999**, *64*, 2966-2968.
[13] C. Venturello, R. D'Aloisio, *The Journal of Organic Chemistry* **1988**, *53*, 1553-1557.
[14] L. Rossi-Fernández, V. Dorn, G. Radivoy, *Beilstein journal of organic chemistry* **2021**, *17*, 519-526.
[15] Y. Tian, E. Jürgens, D. Kunz, *Chemical Communications* **2018**, *54*, 11340-11343.
[16] Z. Zhou, G. Dai, S. Ru, H. Yu, Y. Wei, *Dalton Transactions* **2019**, *48*, 14201-14205.
[17] D. Limnios, C. G. Kokotos, *The Journal of Organic Chemistry* **2014**, *79*, 4270-4276.
[18] E. Mai, C. Schneider, *Chemistry–A European Journal* **2007**, *13*, 2729-2741.
[19] Z.-W. Zhang, H.-B. Li, J. Li, C.-C. Wang, J. Feng, Y.-H. Yang, S. Liu, *The Journal of Organic Chemistry* **2019**, *85*, 537-547.
[20] X. Xiao, D. Lin, S. Tong, H. Mo, *Synlett* **2011**, *2011*, 2823-2826.
[21] M.-M. Lou, H. Wang, L. Song, H.-Y. Liu, Z.-Q. Li, X.-S. Guo, F.-G. Zhang, B. Wang, *The Journal of Organic Chemistry* **2016**, *81*, 5915-5921.
[22] Y. Zhang, X. Yang, H. Tang, D. Liang, J. Wu, D. Huang, *Green Chemistry* **2020**, *22*, 22-27.
[23] J. F. S. Carvalho, M. M. C. Silva, M. L. S. e Melo, *Tetrahedron* **2009**, *65*, 2773-2781.
[24] D. Soto-Castro, R. C. L. Contreras, M. del Socorro Pina-Canseco, R. Santillán, M. T. Hernández-Huerta, G. E. N. Silva, E. Pérez-Campos, S. Rincón, *Steroids* **2017**, *126*, 92-100.
[25] L. D. Dias, R. M. B. Carrilho, C. A. Henriques, G. Piccirillo, A. Fernandes, L. M. Rossi, M. Filipa Ribeiro, M. J. F. Calvete, M. M. Pereira, *Journal of Porphyrins and Phthalocyanines* **2018**, *22*, 331-341.
[26] D. H. Lamparelli, A. Villar-Yanez, L. Dittrich, J. Rintjema, F. Bravo, C. Bo, A. W. Kleij, *Angewandte Chemie* **2023**, *135*, e202314659.
